# Convergent evolution of a metabolic switch between aphid and caterpillar resistance in cereals

**DOI:** 10.1126/sciadv.aat6797

**Published:** 2018-12-05

**Authors:** B. Li, C. Förster, C. A. M. Robert, T. Züst, L. Hu, R. A. R. Machado, J.-D. Berset, V. Handrick, T. Knauer, G. Hensel, W. Chen, J. Kumlehn, P. Yang, B. Keller, J. Gershenzon, G. Jander, T. G. Köllner, M. Erb

**Affiliations:** 1 Institute of Plant Sciences, University of Bern, Bern, Switzerland.; 2 Max Planck Institute for Chemical Ecology, Jena, Germany.; 3 Leibniz Institute of Plant Genetics and Crop Plant Research, Gatersleben, Germany.; 4 Department of Plant and Microbial Biology, University of Zürich, Zürich, Switzerland.; 5 Boyce Thompson Institute, Ithaca, NY, USA.

## Abstract

Tailoring defense responses to different attackers is important for plant performance. Plants can use secondary metabolites with dual functions in resistance and defense signaling to mount herbivore-specific responses. To date, the specificity and evolution of this mechanism are unclear. Here, we studied the functional architecture, specificity, and genetic basis of defense regulation by benzoxazinoids in cereals. We document that DIMBOA-Glc induces callose as an aphid resistance factor in wheat. *O*-methylation of DIMBOA-Glc to HDMBOA-Glc increases plant resistance to caterpillars but reduces callose inducibility and resistance to aphids. DIMBOA-Glc induces callose in wheat and maize, but not in *Arabidopsis*, while the glucosinolate 4MO-I3M does the opposite. We identify a wheat *O*-methyltransferase (TaBX10) that is induced by caterpillar feeding and converts DIMBOA-Glc to HDMBOA-Glc in vitro. While the core pathway of benzoxazinoid biosynthesis is conserved between wheat and maize, the wheat genome does not contain close homologs of the maize DIMBOA-Glc *O*-methyltransferase genes, and *TaBx10* is only distantly related. Thus, the functional architecture of herbivore-specific defense regulation is similar in maize and wheat, but the regulating biosynthetic genes likely evolved separately. This study shows how two different cereal species independently achieved herbivore-specific defense activation by regulating secondary metabolite production.

## Teaser

Maize and wheat independently evolved the capacity to control plant defenses through *O*-methylation of regulatory benzoxazinoids.

## INTRODUCTION

The capacity to resist a large diversity of herbivores is essential for plant fitness in nature and yield in agriculture. Plant resistance strategies are complicated by the fact that herbivores with different lifestyles are susceptible to different types of defenses. Chewing herbivores, for instance, ingest entire leaf parts and are therefore susceptible to plant toxins that accumulate throughout the leaf (*1*, *2*). By contrast, piercing-sucking herbivores, such as aphids, often feed directly from the phloem and are therefore most susceptible to phloem-specific defenses (*3*, *4*). The resulting selection pressure for herbivore-specific deployment of defenses has important consequences for the evolution, physiology, and ecology of plant immunity against herbivores (*5*–*7*).

To mount herbivore-specific defense responses, plants have evolved several regulatory mechanisms. Phytohormonal signaling and cross-talk, for instance, allow plants to finely regulate their induced responses (*8*). In this context, jasmonate (JA) signaling is often associated with induced resistance to chewing herbivores, while salicylate (SA) signaling is associated with resistance to piercing-sucking insects (*9*). However, the picture is likely more complex, as plants use a variety of hormones to form signaling networks that allow for targeted responses against chewing and piercing-sucking herbivores (*8*, *10*).

Apart from hormonal cross-talk, plants can also use secondary metabolites as defensive switches. In maize, the benzoxazinoid 2,4-dihydroxy-7-methoxy-1,4-benzoxazin-3-one-β-d-glucopyranose (DIMBOA-Glc) and its aglucone DIMBOA are positive regulators of callose induction and are associated with increased aphid resistance (*11*), while the DIMBOA-Glc *O*-methylation product 2-(2-hydroxy-4,7-dimethoxy-1,4-benzoxazin-3-one)-β-d-glucopyranose (HDMBOA-Glc) acts as a feeding deterrent against both aphids and chewing herbivores in vitro (*12*, *13*). Upon caterpillar attack, maize plants transform DIMBOA-Glc into HDMBOA-Glc by up-regulating the transcription of three closely related *O*-methyltransferases (OMTs) (ZmBx10–ZmBx12) (*13*, *14*). By contrast, upon aphid attack, the aglucone DIMBOA is secreted into the apoplastic space, and DIMBOA-Glc levels remain high (*11*). Maize DIMBOA-Glc OMTs may therefore act as metabolic switches between caterpillar and aphid resistance. However, the relative contributions of DIMBOA-Glc and HDMBOA-Glc to in planta caterpillar resistance remain to be determined.

Similar to benzoxazinoids in grasses, glucosinolates can regulate callose deposition in *Arabidopsis* (*Arabidopsis thaliana*). 4-Hydroxy-indol-3-yl-methyl glucosinolate (4MO-I3M) is necessary and sufficient to induce callose in response to microbe-associated molecular patterns such as the flagellin peptide flg22 (*15*). Callose deposition depends on the myrosinase PENETRATION 2 (PEN2) and may therefore be elicited by 4MO-I3M breakdown products (*15*). Callose deposition upon infection with fungal and oomycete pathogens is still intact in PEN2- and 4MO-I3M–deficient mutants, suggesting that 4MO-I3M is not strictly required for induced callose deposition in *Arabidopsis* (*16*). Despite their differences, 4MO-I3M and DIMBOA share some structural and biosynthetic similarities, including an aromatic ring bearing an *O*-methoxy group, a glucose moiety and indole as a precursor. Whether the capacity of benzoxazinoids and glucosinolates to regulate callose is conserved across mono- and dicotyledons is unclear (*17*).

A common feature of regulation of herbivore-specific defenses is that these defenses can result in signaling trade-offs. The induction of SA-mediated defenses by piercing-sucking insects, for instance, suppresses JA-dependent volatile defenses against other herbivores (*18*). Furthermore, many early signaling elements, which are required for induced defenses against chewing herbivores, result in susceptibility against piercing-sucking insects (*19*–*21*). Whether secondary metabolite switches also result in similar trade-offs is less understood. In maize, caterpillar feeding increases aphid reproduction in some cultivars, and this effect was mapped to the *ZmBx10–ZmBx12* locus (*22*), lending first support to the hypothesis that the caterpillar-induced conversion of DIMBOA-Glc to HDMBOA-Glc reduces aphid resistance.

While the ecological relevance of herbivore specificity in defense induction is well established, much less is known about the evolution of the underlying mechanisms for that specificity. Analysis of the recent literature suggests that the negative interaction between JA and SA is a conserved trait that was likely already present in the ancestor of angiosperms (*5*). To what extent secondary metabolite switches are evolutionarily conserved is currently unknown. Some secondary metabolites have evolved independently several times (*23*). In general, the rapid evolution and variation in plant secondary metabolites (*24*, *25*) may favor convergent evolution of secondary metabolite switches mediating specific herbivore resistance.

In this study, we characterized the specificity, functional architecture, and genetic basis of aphid and caterpillar resistance in wheat. On the basis of current knowledge on caterpillar and aphid resistance in maize, we focused on benzoxazinoids as direct and indirect regulators of resistance. The core benzoxazinoid pathway is conserved among different cereals and likely to be of monophyletic origin within the Poaceae (*26*). Furthermore, the induction of HDMBOA-Glc at the expense of DIMBOA-Glc upon caterpillar attack occurs in both wheat and maize (*12*, *27*). We therefore hypothesized that the switch between aphid and caterpillar resistance should be conserved between the two species as well. To test this hypothesis, we first explored the functional architecture of benzoxazinoid-dependent defense and resistance by overexpressing a maize DIMBOA-Glc OMT in wheat. This approach allowed us to explore the importance of HDMBOA-Glc accumulation for caterpillar resistance in vivo and to quantify the contribution of DIMBOA-Glc methylation to caterpillar-induced aphid susceptibility. To evaluate the specificity of callose regulation by benzoxazinoids, we conducted complementation experiments with benzoxazinoids and glucosinolates in wheat and *Arabidopsis*. Last, we analyzed the genetic architecture of the benzoxazinoid switch by transcriptional profiling, heterologous expression, and genome-based phylogenetic analyses.

## RESULTS

### Influence of DIMBOA-Glc *O*-methylation on wheat defense

**Fig. 1:**
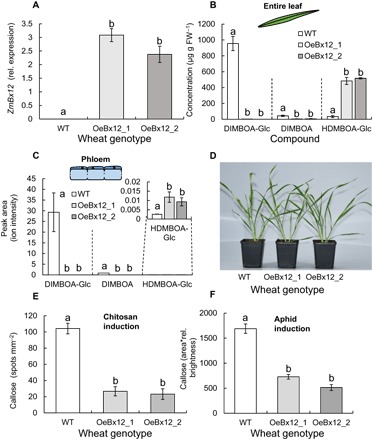
Phenotype of *ZmBx12*-overexpressing wheat lines. (**A**) Relative (rel.) expression of the maize DIMBOA-Glc OMT *ZmBx12* in wild-type (WT) and *ZmBx12*-overexpressing plants (*n* = 3). (**B**) Major benzoxazinoids in the leaves of the different lines (*n* = 5). FW, fresh weight. (**C**) Major benzoxazinoids in the phloem of the different lines (*n* = 3 to 4). (**D**) Representative photograph of WT and transgenic lines. (**E**) Chitosan-induced callose deposition (*n* = 9 to 12). (**F**) Aphid-induced callose deposition (*n* = 23 to 39). Because of irregular shapes and brightness of callose induction spots following aphid attack, area*rel. brightness were used to assess callose deposition. Different letters indicate significant differences between wheat lines [analysis of variance (ANOVA) followed by Holm-Sidak post hoc tests, *P* < 0.05]. Photo credit for (D): B. Li and T. Züst, University of Bern.

To explore the role of DIMBOA-Glc *O*-methylation in wheat, we generated transgenic plants harboring the DIMBOA-Glc OMT gene *ZmBx12* from maize under the control of a constitutive maize *UBIQUITIN1* promoter. Experiments were carried out using two T2 homozygous transgenic lines (OeBx12_1 and OeBx12_2), which constitutively expressed *ZmBx12* (Fig. 1A). Compared with wild-type plants, OeBx12 leaves accumulated high amounts of HDMBOA-Glc at the expense of DIMBOA-Glc and DIMBOA, which were almost entirely depleted (Fig. 1B). The HDMBOA-Glc breakdown product MBOA also accumulated in higher amounts in OeBx12 lines, while HMBOA-Glc, which is likely formed from DIMBOA-Glc or DIBOA-Glc, was present in lower concentrations (fig. S1). Levels of the other benzoxazinoids in the leaves did not change significantly, apart from a slight increase in DIM_2_BOA-Glc in one of the OeBx12 lines (fig. S1). Similar patterns were found in phloem sap collected through aphid stylectomy (Fig. 1C). While DIMBOA-Glc and DIMBOA were dominant in the phloem of wild-type plants, OeBx12 lines accumulated high amounts of HDMBOA-Glc at the expense of DIMBOA-Glc and DIMBOA (Fig. 1C). OeBx12 plants grew similar to their wild-type counterparts (Fig. 1D) and accumulated similar amounts of sugars, starch, and soluble protein in their leaves (fig. S1). We also did not find any consistent changes in free amino acids in the leaves and phloem of wild-type and OeBx12 plants (fig. S1). Higher amounts of ferulic acid were observed in OeBx12 plants compared with wild-type plants (fig. S2). Furthermore, OeBx12 plants displayed less callose induction following treatment with the fungal elicitor chitosan (Fig. 1E) and aphid infestation (Fig. 1F). Thus, the functional architecture of benzoxazinoid regulation of callose seems to be similar in wheat and maize.

**Fig. 2:**
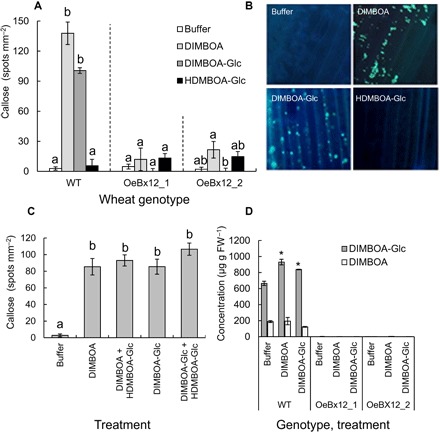
Callose suppression through DIMBOA-Glc *O*-methylation. (**A**) Induction of callose in WT and *ZmBx12*-overexpressing lines after infiltration with DIMBOA, DIMBOA-Glc, or HDMBOA-Glc (*n* = 4 to 11). (**B**) Representative photographs of callose induction in WT plants after aniline blue staining. (**C**) Callose induction in WT plants treated with different benzoxazinoid mixtures (*n* = 5 to 11). (**D**) Concentration of DIMBOA-Glc (dark gray bars) and DIMBOA (light gray bars) in the leaves of WT and transgenic plants infused with DIMBOA-Glc or DIMBOA (*n* = 3). Different letters and asterisks indicate significant differences between treatments within lines (ANOVA followed by Holm-Sidak post hoc tests, *P* < 0.05). Photo credit for (B): B. Li, University of Bern.

To better understand the relationship between benzoxazinoids and callose induction, we infiltrated wild-type and OeBx12 plants with DIMBOA, DIMBOA-Glc, and HDMBOA-Glc. DIMBOA and DIMBOA-Glc treatments induced significant callose deposition in the leaves of wild-type plants, while HDMBOA-Glc had no effect (Fig. 2A). Unexpectedly, complementation of OeBx12 plants with DIMBOA or DIMBOA-Glc did not result in any callose induction (Fig. 2A). We devised two hypotheses to explain this result. First, we postulated that the high levels of HDMBOA-Glc in OeBx12 plants may inhibit callose induction. To test this hypothesis, we infused wild-type leaves with combinations of DIMBOA, DIMBOA-Glc, and HDMBOA-Glc. Co-infusion of HDMBOA-Glc did not suppress callose induction by DIMBOA and DIMBOA-Glc (Fig. 2C). As an alternative hypothesis, we tested whether the infused DIMBOA and DIMBOA-Glc are rapidly metabolized in OeBx12 lines. Infusion of DIMBOA and DIMBOA-Glc increased DIMBOA-Glc levels in wild-type plants, but not in OeBx12 lines (Fig. 2D), where levels remained constantly low. The levels of two likely metabolic products, HDMBOA-Glc and MBOA, were not significantly increased in the infiltrated leaves in any of the lines upon DIMBOA or DIMBOA-Glc infiltration (fig. S3). These results suggest that either rapid transformation to yet unidentified, inactive catabolites or rapid transport of HDMBOA-Glc or MBOA into systemic tissues may have prevented the induction of callose by DIMBOA and DIMBOA-Glc in OeBx12 lines.

### Specificity of callose induction by benzoxazinoids and glucosinolates

To better understand the specificity of DIMBOA and DIMBOA-Glc as callose inducers, we infused the metabolites into *Arabidopsis* leaves. As a cross-comparison, we infused the glucosinolate 4MO-I3M, which is known to induce callose in *Arabidopsis* (*15*), into *Arabidopsis* and wheat. 4MO-I3M, but not DIMBOA or DIMBOA-Glc, induced callose in *Arabidopsis* (fig. S4A). 4MO-I3M did not induce callose in wheat (fig. S4B). Overall, we did not detect any callose induction in cross-infusions. Thus, the induction of callose by secondary metabolites is specific for the respective plant species.

### Influence of DIMBOA-Glc *O*-methylation on caterpillar and aphid resistance

**Fig. 3:**
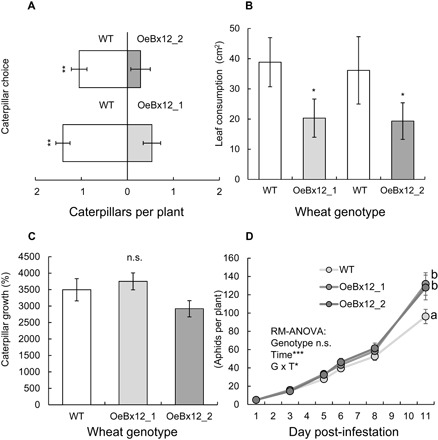
DIMBOA-Glc *O*-methylation changes aphid and caterpillar resistance in opposite directions. (**A**) Preference of *S. littoralis* caterpillars for WT or *ZmBx12*-overexpressing plants (*n* = 15 to 21). (**B**) Leaf damage in a choice situation (*n* = 8). Asterisks indicate significant differences between lines (one-sample *t* tests on pairwise differences, *P < 0.05, **P < 0.01, ***P < 0.001). (**C**) Caterpillar growth in a no-choice experiment. (**D**) *S. avenae* aphid reproduction on different lines. Different letters indicate differences between plant lines (repeated-measures ANOVA followed by Holm-Sidak post hoc tests, *P* < 0.05). Significance levels for repeated-measure ANOVA factors are shown. n.s., not significant.

In maize, high DIMBOA-Glc levels are required for aphid resistance (*13*), while HDMBOA-Glc has been shown to act as a deterrent for caterpillars in vitro (*12*). To test whether DIMBOA-Glc *O*-methylation affects aphid and caterpillar resistance in wheat, we conducted a series of preference and performance tests. When given a choice, *Spodoptera littoralis* caterpillars preferred to feed on wild-type plants rather than on OeBx12 lines (Fig. 3A). Furthermore, they consumed more leaf biomass on wild-type plants (Fig. 3B). Caterpillar growth in no-choice experiments was not significantly affected (Fig. 3C). *Sitobion avenae* aphids, on the other hand, grew better on OeBx12 than wild-type plants (Fig. 3D). Thus, DIMBOA-Glc *O*-methylation increases caterpillar resistance through antixenosis but decreases aphid resistance in the form of antibiosis. To test whether DIMBOA-Glc *O*-methylation affects resistance against two common fungal pathogens, we quantified infection by powdery mildew (*Blumeria graminis* f. sp. *tritici*) and leaf rust (*Puccinia recondita* f. sp. *tritici*). *OeBx12* expression did not affect wheat resistance against these pathogens (fig. S5).

**Fig. 4:**
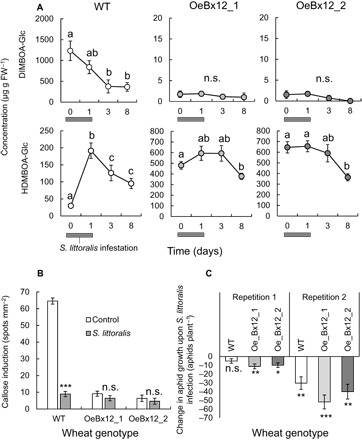
DIMBOA-Glc *O*-methylation mediates a trade-off between induced caterpillar and aphid resistance. (**A**) Changes in major benzoxazinoids in WT and *ZmBx12*-overexpressing lines at different time points following 24 hours of *S. littoralis* attack (*n* = 5 to 7). Different letters indicate significant differences in concentrations over time (ANOVA followed by Holm-Sidak post hoc tests, *P* < 0.05). (**B**) Callose inducibility in control and *S. littoralis*–attacked wheat plants (*n* = 12 to 20). Asterisks indicate significant differences between treatments (two-way ANOVA followed by Holm-Sidak post hoc tests, *P < 0.05, **P < 0.01, ***P < 0.001). (**C**) Aphid performance on *S. littoralis–*attacked wheat plants relative to nonattacked controls (*n* = 9 to 11). Results from two repetitions of the experiments are shown side by side. Stars indicate a significant reduction of aphid growth induced by *S. littoralis* caterpillar feeding relative to *S. littoralis*–unattacked plants (two-way ANOVA followed by Holm-Sidak post hoc tests.

Caterpillar attack increases DIMBOA-Glc *O*-methylation, and the resulting suppression of callose through DIMBOA-Glc depletion may weaken aphid resistance. To test this hypothesis, we first profiled changes in benzoxazinoids in the leaves of wild-type and OeBx12 plants, which were induced by *S. littoralis* for 24 hours. In wild-type plants, HDMBOA-Glc levels increased sixfold within 24 hours, and DIMBOA-Glc levels dropped threefold within 72 hours (Fig. 4A). By contrast, no significant changes were observed in OeBx12 plants, apart from a slight reduction in HDMBOA-Glc over time (Fig. 4A). In contrast to caterpillar attack, infestation with *S. avenae* aphids did not change DIMBOA-Glc, DIMBOA, or HDMBOA-Glc levels in wheat leaves (fig. S6). Chitosan-induced callose deposition was significantly suppressed upon *S. littoralis* attack in wild-type plants. By contrast, no changes in callose inducibility were observed upon *S. littoralis* attack in OeBx12 plants (Fig. 4B). These results suggest that the *S. littoralis*–mediated suppression of DIMBOA-Glc leads to a reduction in callose inducibility.

To test whether DIMBOA-Glc *O*-methylation affects the interaction between caterpillars and aphids on the same plant, aphids were left to grow on *S. littoralis*–induced leaves of wild-type and OeBx12 plants in two different experiments. Overall, *S. littoralis* attack had a negative effect on aphid growth. The magnitude of this effect varied between experiments (Fig. 4C). In both experiments, the negative influence of *S. littoralis* attack on aphid performance was slightly more pronounced in OeBx12 plants than in wild-type plants (Fig. 4, C and D). Thus, the absence of callose suppression in OeBx12 plants is associated with a slight accentuation of caterpillar-induced resistance to aphids.

### Identification of the wheat DIMBOA-Glc OMT TaBX10

As the core pathway of benzoxazinoid biosynthesis is conserved between wheat and maize (*26*), we hypothesized the wheat DIMBOA-Glc OMT might be an ortholog of maize *ZmBx10–ZmBx12* or *ZmBx14* (*14*, *28*). However, initial BLAST analysis revealed no orthologs of these genes in the wheat genome. We therefore sequenced the transcriptomes of *S. littoralis*–damaged and *S. littoralis*–undamaged wheat leaves to identify potential herbivore-induced *OMT* genes (fig. S7). Mapping of the obtained Illumina reads to the wheat gene model version 2.2 (*29*) and subsequent analysis of digital gene expression (EDGE) identified eight *OMT* gene candidates that were differentially expressed in the two treatments (fig. S7B and table S1). While none of the eight genes were homologous to *ZmBx10/11/12/14*, five of them showed a moderate sequence similarity to *ZmBx7*, an OMT involved in the methylation of hydroxyl groups on the aromatic ring of the benzoxazinoid core structure (fig. S8) (*28*, *30*). The remaining three candidates were only distantly related to benzoxazinoid OMTs and grouped together with SABATH methyltransferase genes from maize, suggesting a function as benzenoid carboxyl methyltransferases (*31*, *32*). Sequence comparison of the five wheat *OMT* genes similar to *ZmBx7* revealed that only two of them encoded full-length OMT proteins (fig. S9). These two genes, *Traes 4AL C467B516F* and *Traes 2BL C467B516F*, showed 100% nucleotide identity to each other. Quantitative reverse transcription polymerase chain reaction (qRT-PCR) analysis confirmed that they were strongly up-regulated after herbivory (fig. S7C). Construction of a de novo transcriptome based on the Illumina reads from *S. littoralis*–damaged wheat leaves revealed no further herbivore-induced *ZmBx10/11/12/14*-like *OMT* genes in comparison to those found in the gene model version 2.2 (fig. S10).

**Fig. 5:**
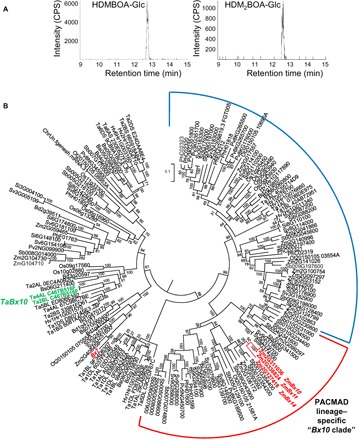
Identification and characterization of TaBX10 as a functional DIMBOA-Glc OMT. (**A**) Recombinant TaBX10 methylates DIMBOA-Glc and DIM_2_BOA-Glc. The enzyme was heterologously expressed in *E. coli*, purified, and incubated with a mixture of DIMBOA-Glc and DIM_2_BOA-Glc. Enzyme products were analyzed using liquid chromatography–tandem mass spectrometry (LC-MS/MS). CPS, counts per second (electron multiplier). (**B**) Phylogenetic tree of Poaceae *OMT* genes similar to *Bx7*. Maize *Bx7,10,11,14* and wheat *TaBx10* are shown in red and green, respectively. The tree represents a subclade of a larger Poaceae *OMT* tree that is given in fig. S13. The tree was inferred by using the maximum likelihood method based on the Tamura 3-parameter model. Bootstrap values are shown next to each node. The tree is drawn to scale, with branch lengths measured in the number of substitutions per site. Zm, *Zea mays*; Sb, *Sorghum bicolor*; Sv, *Setaria viridis*; Si, *S. italica*; Pv, *Panicum virgatum*; Ph, *P. hallii*; Ot, *Oropetium thomaeum*; Ta, *T. aestivum*; Hv, *Hordeum vulgare*; Bd, *Brachypodium distachyon*; Bs, *B. stacei*; Os, *Oryza sativa*. The PACMAD lineage–specific *Bx10* clade is marked in red, and its sister clade is marked in blue.

To characterize the enzymatic activity of the OMT protein encoded by Traes 4AL C467B516F/Traes 2BL C467B516F, the complete open reading frame, designated as *TaBx10*, was amplified from complementary DNA (cDNA) made from herbivore-damaged wheat leaves and heterologously expressed in *Escherichia coli*. In the presence of the OMT cosubstrate *S*-adenosyl-l-methionine, purified recombinant TaBX10 was able to convert DIMBOA-Glc and DIM_2_BOA-Glc into HDMBOA-Glc and HDM_2_BOA-Glc, respectively (Fig. 5A). Maize ZmBX10 was used as positive control and showed exclusively DIMBOA-Glc OMT activity, as previously reported (*13*, *28*). No product formation was observed when the substrates were incubated with an empty vector control (fig. S11). *TaBx10* expression was similarly induced by *S. littoralis* in wild-type and OeBX12 lines (fig. S12).

### Independent evolution of benzoxazinoid OMT activity in maize and wheat

Maize and wheat belong to the Panicoideae (PACMAD lineage) and Pooideae (BEP lineage), respectively, two major grass subfamilies that diverged 50 to 70 Ma ago (*33*). Our initial sequence comparison of maize and wheat *OMT* genes revealed that the two *TaBx10* gene copies have only 54 to 58% nucleotide sequence identity to *ZmBx10/11/12/14* and are not directly related to the *ZmBx10/11/12/14* gene cluster in maize (fig. S8), suggesting independent evolution of DIMBOA-Glc OMT activity in the Panicoideae and Pooideae. To study this evolutionary scenario in more detail, we extracted full-length *OMT* gene sequences with similarity to *Bx7* from all Poaceae genomes available in the Phytozome database and included them into our phylogenetic analysis. The resulting phylogenetic tree showed that maize *ZmBx10/11/12/14* clustered within a well-defined clade (PACMAD lineage–specific *Bx10* clade), which comprised exclusively genes from Panicoideae and Chloridoideae species (Fig. 5B and fig. S13). In contrast, the sister clade contained genes from various Pooideae/Chloridoideae and Panicoideae species including wheat and maize. This indicates that the ancestors of the PACMAD lineage–specific *Bx10* clade and its sister clade must have existed before the separation of the Panicoideae/Chloridoideae and Pooideae and that the *Bx10* clade ancestor was probably lost early in the evolution of the Pooideae. The fact that the two *TaBx10* gene copies grouped together with other Pooideae and Panicoideae genes in a clade that was separated from the *Bx10* clade and its sister clade (Fig. 5B) confirms our initial hypothesis of independent evolution of DIMBOA-Glc OMT activity within the grasses.

### Independent evolution of benzoxazinoid and glucosinolate OMT

To test whether benzoxazinoid *OMT* genes are related to glucosinolate *OMT* genes, we included indole glucosinolate *O*-methyltransferase 1 (IGMT1) and IGMT2, two recently identified OMTs from *Arabidopsis* that catalyze the methylation of 4-hydroxy-indol-3-yl-methyl glucosinolate (4OH-I3M) to 4MO-I3M (*37*), into our phylogenetic analysis. As expected, *IGMT1* and *IGMT2* grouped together with other *Arabidopsis OMT* genes and were separated from *TaBx10* or *ZmBx10/11/12/14* (fig. S14), suggesting independent evolution of benzoxazinoid and glucosinolate OMT activity.

## DISCUSSION

Plants can use secondary metabolites to deploy herbivore-specific defense responses. This study illustrates that a single, herbivory inducible methylation step serves as a switch and trade-off point between aphid and caterpillar resistance in wheat. Unexpectedly, while the core biosynthesis pathway and the functional architecture of the defense switch are similar in wheat and maize, the responsible enzymes have evolved independently from each other. Below, we discuss these results from physiological, ecological, and evolutionary points of view.

The role of benzoxazinoids, including the methylation of DIMBOA-Glc to HDMBOA-Glc, in plant-herbivore interactions has been studied in detail in maize (*12*–*14*, *22*, *28*, *34*). In vitro experiments with DIMBOA and HDMBOA-Glc suggested that HDMBOA-Glc may be more potent as a feeding deterrent, as it is rapidly deglycosylated and converted into unstable breakdown products (*35*), which precludes its detoxification by reglycosylation (*12*). Our results support this hypothesis by showing that the overproduction of HDMBOA-Glc renders plants less attractive and reduces leaf damage by *S. littoralis*. Apparently, HDMBOA-Glc acts as a strong antixenotic rather than an antibiotic, as caterpillar growth was not reduced in the transgenic lines. How caterpillars avoid negative consequences of HDMBOA-Glc on their digestive physiology remains to be determined. The increased levels of ferulic acid in HDMBOA-Glc–overproducing lines suggest that other secondary metabolites may be regulated by DIMBOA-Glc *O*-methylation. While the mechanisms behind this phenomenon remain unclear, it is possible that the increased levels of other defenses in HDMBOA-Glc–overproducing lines may have contributed to the reduction in caterpillar feeding.

Although HDMBOA-Glc is also toxic to aphids (*14*), aphids grow better on maize cultivars with high HDMBOA-Glc levels and low DIMBOA-Glc levels, which coincides with lower callose inducibility in these genotypes (*14*). Together with the fact that caterpillar attack induces HDMBOA-Glc production and depletes DIMBOA-Glc reserves (*12*), this led to the hypothesis that caterpillar feeding should induce aphid susceptibility by suppressing callose accumulation (*22*). Our experiments not only confirm this hypothesis but also demonstrate that callose inducibility only partially explains the interaction between *S. littoralis* and *S. avenae* in wheat. Aphid performance was decreased on caterpillar-attacked leaves even in the absence of DIMBOA-Glc depletion, suggesting that other inducible defenses increase aphid resistance. Caterpillar attack leads to substantial transcriptional and metabolic reprogramming of a plant’s primary and secondary metabolism (*36*–*39*), and it is therefore not unusual that physiological changes other than benzoxazinoid-dependent callose deposition contribute to the overall outcome of the interaction between caterpillars and aphids. In maize, genetic mapping revealed substantial variation and multiple quantitative trait loci associated with caterpillar-induced resistance and susceptibility against aphids (*22*), which may provide a path toward the functional characterization of these additional factors.

The resistance of wheat to fungal pathogens has been associated with callose deposition (*40*). In this context, it is unexpected that the suppressed callose inducibility in Oe_Bx12 lines did not affect their resistance to two fungal pathogens. We propose three hypotheses to explain this result. First, it is possible that the fungi are more susceptible to HDMBOA-Glc than DIMBOA-Glc, which may have counterbalanced resistance in the transgenic lines. Second, the higher production of other defenses in HDMBOA-Glc–overproducing lines may have offset the effects of reduced callose deposition. Third, the two pathogens may suppress or avoid callose induction. In maize, fungal pathogen attack leads to a marked increase of HDMBOA-Glc levels (*27*, *41*), and it is possible that such an induction, similar to caterpillar attack, suppresses callose inducibility in wheat and thereby allows the fungus to suppress effective defenses.

**Fig. 6:**
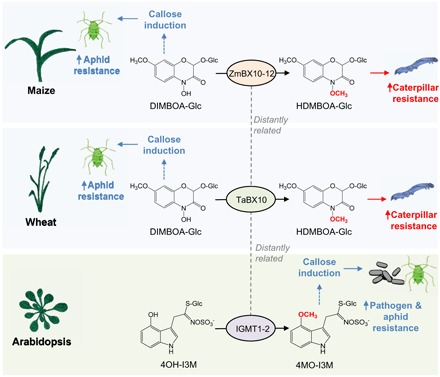
Independently evolved *O*-methylation of benzoxazinoids and glucosinolates regulates defense and resistance. DIMBOA-Glc is required for callose induction in wheat and maize. Methylation of DIMBOA-Glc to HDMBOA-Glc reduces the DIMBOA-Glc pool and subsequently suppresses callose formation. The responsible OMTs, TaBX10 and ZmBX10–ZmBX12, evolved independently from each other. HDMBOA-Glc repels caterpillars, while DIMBOA-Glc reduces aphid growth, most likely by promoting callose formation. In *Arabidopsis*, methylation of the glucosinolate 4OH-I3M by IGMT1 and IGMT2 leads to the formation of 4MO-I3M, which is required for callose deposition in this species. 4MO-I3M increases aphid and pathogen resistance, most likely by promoting callose formation. IGMT1 and IGMT2 are only distantly related to cereal benzoxazinoid OMTs.

Overall, our experiments and the current literature reveal that the architecture of benzoxazinoid-dependent defense regulation is notably similar in maize and wheat (Fig. 6). In both species, DIMBOA-Glc is generally the dominant constitutively produced benzoxazinoid, and HDMBOA-Glc is strongly induced and becomes dominant upon caterpillar and pathogen attack (*12*, *27*, *41*). Furthermore, callose deposition is induced by DIMBOA and DIMBOA-Glc, but not by HDMBOA-Glc (*11*). Genetic analyses revealed that the core biosynthetic pathway that converts indole-3-glycerol phosphate to DIBOA-Glc via several intermediate steps is largely conserved between wheat and maize (*26*). The fact that the DIMBOA-Glc *OMT* genes in wheat and maize are analogs rather than orthologs is an unexpected finding in this context. Phylogenetic analysis suggests that the capacity to produce HDMBOA-Glc and to use the DIMBOA-Glc OMT as a regulatory switch between aphid and caterpillar resistance evolved independently in maize and wheat. The functional validation of the wheat DIMBOA-Glc OMT TaBX10 in planta would shed further light on its contribution to HDMBOA-Glc biosynthesis relative to other potential wheat OMTs. Although HDMBOA-Glc has also been found in Job’s tears (*Coix lacryma-jobi*) (*42*), nothing is known about HDMBOA-Glc production in the other benzoxazinoid-producing plant species. Investigating and comparing the evolutionary relationships and defense regulation of benzoxazinoids in additional species are exciting prospects of this work.

Comparing callose induction of benzoxazinoids and glucosinolates revealed a high degree of specificity. While the benzoxazinoid-producing wheat responds strongly to DIMBOA and DIMBOA-Glc, it does not respond to the glucosinolate 4MO-I3M. Conversely, the glucosinolate-producing *Arabidopsis* responds strongly to 4MO-I3M, but not to DIMBOA or DIMBOA-Glc. As expected, the OMTs involved in the production of 4MO-I3M (*43*) and HDMBOA-Glc (*14*) are only distantly related to each other. Thus, callose regulation by benzoxazinoids and glucosinolates as illustrated in Fig. 6 does not proceed via the same mechanism, and it is likely that the two phenomena evolved independently from each other. Glucosinolate hydrolysis is required for callose induction in *Arabidopsis* (*15*), while DIMBOA seems to induce callose in the absence of any measurable increase in known breakdown products. As glucosinolate breakdown products are structurally distinct from benzoxazinoids and their catabolites, they would likely require a different mechanism of perception to be integrated into defense signaling (Fig. 6). Nevertheless, it is theoretically possible that the signaling cascades elicited by benzoxazinoids and glucosinolate share a common ancestor and diverged since the split of the mono- and dicotyledons 140 Ma ago (*44*). A better understanding of the molecular and genetic basis of callose regulation by benzoxazinoids and glucosinolates will be required to disentangle these hypotheses.

Convergence is an important force in evolution (*45*), and plants provide many fascinating examples in this context (*46*). Caffeine biosynthesis, for instance, evolved at least five times within the flowering plants, partially by co-opting enzymes with different ancestral functions (*47*). In general, plant secondary metabolism can evolve rapidly due to enzyme promiscuity and frequent gene duplications (*25*, *48*, *49*). Secondary metabolites may thus allow plants to rapidly and specifically adapt their defense signaling systems to different herbivore and pathogen pressures. The finding that maize and wheat co-opted the same secondary metabolite pathway to specifically regulate defenses against aphids and caterpillars is suggestive of an important adaptive role of this form of herbivore-specific defense regulation.

## MATERIALS AND METHODS

Note that the numbers of replicates for the individual experiments are provided in the respective figure legends.

### Plant and insect material

The wheat (*Triticum aestivum*) CYMMIT breeding line Bobwhite SH 98 26 (*50*) was used in this study. Plants were grown in a climate-controlled room with 16 hours photoperiod, 300 μmol m^−2^ s^−1^ of photosynthetically active radiation, and a temperature cycle of 22°C/18°C (light/dark). *S. littoralis* caterpillars were provided by T. Turlings (FARCE Laboratory, University of Neuchâtel, Switzerland) and were kept on artificial diet at 22°C. The English grain aphid (*S. avenae*) was obtained from Andermatt Biocontrol AG (Switzerland) and maintained on barley seedlings in a growth chamber with 16:8 light/dark photoperiod at 24°C.

### Generation of *ZmBx12*-expressing transgenic wheat

The complete open reading frame of *ZmBx12-CML322* was amplified with the primers ZmBx12_SpeI-fwd (GACTAGTCATGCAAGAGAGCAGTAGC) and ZmBx12_EcoRI-rev (GGAATTCCTCAAGGATAGACCTCGATGATGA) from the previously described vector pASK-IBA37plus::*ZmBx12* (*13*). The amplification product was digested with Spe I and Eco RI (Fast digest, Thermo Fisher Scientific) and subcloned into the linearized vector pUbi-AB downstream of the maize UBIQUITIN 1 promoter (*ZmUbi1*) (*51*). The resulting construct pUbi-AB::*ZmBx12* was digested with Sfi I (Fast digest, Thermo Fisher Scientific), and the digestion products were separated by gel electrophoresis. The fragment containing *ZmUbi1::ZmBx12* was subsequently extracted from the gel, purified, and subcloned into the binary vector p6i-d35S (*51*) that was linearized by Sfi I digestion. The insertion of *ZmUbi1::ZmBx12* into p6i-d35S was confirmed by a control digestion with Kpn I and Hind III (Fast digest, Thermo Fisher Scientific). The binary vector was transferred to the *Agrobacterium tumefaciens* strain AGL1 and used to inoculate immature Bobwhite SH 98 26 embryos. The generation of transgenic plants and their molecular analysis for transgene integration were conducted as previously described (*52*), with the exception of Southern blot analysis, which could not be completed successfully. The transgenic lines may therefore harbor single or multiple insertions of *ZmUbi1::ZmBx12*. Segregation analysis was conducted on T2 plants by using the ratio of DIMBOA-Glc to HDMBOA-Glc as a marker. T2 seeds of two homozygous T1 plants harboring at least one highly expressed, complete insertion of *ZmUbi1::ZmBx12* were used for biological experiments.

### RNA extraction, cDNA synthesis, and quantitative real-time PCR

Total RNA was extracted from leaves of 14-day-old wild-type and *ZmBx12*-overexpressing plants using an RNA isolation kit (Thermo Fisher Scientific, USA). Two microgram of total RNA was used for reverse transcription with SuperScript II reverse transcriptase (Invitrogen, USA). The cDNA samples were diluted to 2 to 8 ng/μl. Triplicate quantitative assays were performed on 5 μl of each cDNA dilution with the Kapa SYBR Fast qPCR Master Mix (Sigma, USA) and a LightCycler96 detection system (Roche, Switzerland) according to the manufacturer’s protocol. The relative quantification method (Delta-Delta CT) was used to evaluate quantitative variation between the replicates examined. The amplification of *T*. *aestivum* *Tubulin* was used as an internal control to normalize all data, and primers for *Tubulin* were ATCTGGTGCGGGTAACAA (forward) and AAGTGGAGGCGAGGGAAT (reverse). Gene-specific primers for *ZmBx12* were ATGGCACTCATGCAAGAGAGC (forward) and TCAAGGATAGACCTCGATGATG (reverse).

For qRT-PCR analysis of *TaBx10*, RNA was extracted using the InviTrap Spin Plant RNA Mini Kit (Stratec, Berlin, Germany) according to the manufacturer’s instructions. cDNA was prepared from 1 μg of deoxyribonuclease-treated RNA using SuperScript III reverse transcriptase (Invitrogen, Carlsbad, CA, USA) and diluted 1:10 with water. Primers for *TaBx10* were TCCCCGATGGTGGGCA (forward) and GGTGGTGTCCCAGAACGTG (reverse). Primer specificity was confirmed by agarose gel electrophoresis, melting curve analysis, and standard curve analysis and by sequence verification of cloned PCR amplicons. Primer pair efficiency (98.5%) was determined using the standard curve method with twofold serial dilutions of cDNA. Samples were run in triplicate using the Brilliant III Ultra-Fast SYBR Green QPCR Master Mix (Agilent Technologies, Santa Clara, CA, USA). The following PCR conditions were applied for all reactions: initial incubation at 95°C for 3 min, followed by 40 cycles of amplification (95°C for 10 s, 60°C for 10 s). All samples were run on the same PCR machine (CFX Connect Real-Time System; Bio-Rad Laboratories, Hercules, CA, USA) in an optical 96-well plate. Six biological replicates for each treatment were analyzed as triplicates.

### Collection of wheat fascicular phloem

Leaves of 14-day-old wheat plants were fixed inside a flat tray with double-sided adhesive tape. About 10 to 20 adult aphids (*S. avenae*) were placed onto the upper surface of each leaf and allowed to feed overnight. After cutting the aphid stylets with a microcautery device (CF-50, Syntech) according to Fisher and Frame (*53*), the flat tray with the fixed leaves and cut stylets was flooded with silicon oil (M 200, Roth) to prevent evaporation of the exudates. Twenty-four hours later, the phloem sap was collected under silicon oil using a microcapillary connected to a small syringe via a silicone tube with a side valve. Depending on the exudation time of the severed stylets, the sample amount was up to 1.4 μl per stylet during 24 hours. The samples of each plant were pooled and stored at −20°C. For LC-MS/MS analysis of benzoxazinoids (see below), phloem samples were diluted 1:7 with methanol:water (1:1, v/v).

### Extraction and analysis of benzoxazinoids, phenolic acids, amino acids, proteins, sugars, and starch

Plant leaf tissue was ground into fine powder under liquid nitrogen. The frozen powder was weighed, and extraction buffer (50% methanol, 0.1% formic acid) was added immediately (1 mg, 10 μl). The plant sample was vortexed for 30 s and then centrifuged at 11,000*g* for 20 min at 4°C. The supernatant was transferred into a new Eppendorf tube and centrifuged once more to remove all particles.

Benzoxazinoid content was analyzed with an Acquity UPLC (Waters, USA) coupled to an ultraviolet (UV) detector and a QDa mass spectrometer (Waters, USA) using an Acquity BEH C18 column (2.1 mm by 100 mm, 1.7 μm; Waters, USA). The temperatures of the autosampler and column were set to 15° and 40°C, respectively. The mobile phase consisted of 99% water, 1% acetonitrile, and 0.1% formic acid (A), and acetonitrile and 0.1% formic acid (B). Flow rate was set to 0.4 ml min^−1^ with 97% A and 3% B. The injection volume was 5 μl. The elution profile was: 0 to 9.65 min, 3 to 16.4% B; 9.65 to 13 min, 16.4 to 100% B; and 13.1 to 15 min at 100% B, followed by 2-min column reconditioning at 3% B. The extracted UV trace at 275 nm was used for benzoxazinoid quantification. The following extracted ion chromatograms were used for quantification with a mass window of ±0.01 D: 164 mass-to-charge ratio (*m/z*) for DIMBOA [retention time (RT), 5.62 min], 372 *m/z* for DIMBOA-Glc (RT, 5.64 min), 432 *m/z* for HDMBOA-Glc (RT, 8.19 min), 402 *m/z* for DIM_2_BOA (RT, 5.81 min), 356 *m/z* for HMBOA-Glc (RT, 5.28 min), 342 *m/z* for DIBOA-Glc (RT, 4.29 min), 462 *m/z* HDM_2_BOA-Glc (RT, 8.32 min), 164 *m/z* for MBOA (RT, 8.17 min), and 372 *m/z* for MOA-Glc (RT, 5.31 min). Benzoxazinoid concentrations were determined by external calibration curves obtained from purified DIMBOA-Glc, DIMBOA, and HDMBOA-Glc standards. Five calibration points (5, 10, 50, 150, and 200 μg/ml) were used.

Quantification of phenolic acids (chlorogenic acid, p-caffeic acid, p-coumaric acid, ferulic acid, and sinapic acid) was performed on an Acquity UPLC coupled to a Xevo G2 XS Q-TOF time-of-flight mass spectrometer (Waters, USA) equipped with an electrospray ionization (ESI) source. The compounds were separated on an Acquity BEH C18 ultra performance liquid chromatography (UPLC) column (2.1 mm by 50 mm internal diameter, 1.7 μm particle size). Water (0.1% formic acid) and acetonitrile (0.1% formic acid) were used as mobile phases A and B. The gradient profile was as follows: 0 to 1 min, 95% A in B; 1 to 5 min, 81% A in B; 5 to 5.2 min, 1% A in B; 5.2 to 6.5 min, 1% A in B; 6.6 to 8 min, 95% A. The mobile phase flow rate was 0.4 ml/min. The column temperature was maintained at 40°C, and the injection volume was 1 μl. The quadrupole orthogonal acceleration–time-of-flight (Q-TOF) was operated in ESI negative mode, and data were acquired in scan range (50 to 1200 *m/z*) using a cone voltage of 20 V. The elution order was as follows: 2.09 min, chlorogenic acid; 2.22 min, p-caffeic acid; 3.18 min, p-coumaric acid; 3.83 min, ferulic acid; and 4 min, sinapic acid. Chromatograms were acquired in MS^E^ mode using a collision energy ramping of 10 to 30 V. Quantification of the compounds was performed on the basis of their exact masses: chlorogenic acid, 353.0872 *m/z*; p-caffeic acid, 179.0344 *m/z*; p-coumaric acid, 163.0395 *m/z*; ferulic acid, 3.83 min and 193.050 *m/z*; and sinapic acid, 4 min and 223.0606 *m/z*. Calibration solutions containing the different phenolic acids were prepared between 0 and 5 μg ml^−1^ to establish linear regressions.

To determine amino acid concentrations in wheat phloem, phloem samples were diluted 1:7000 with methanol:water (1:1, v/v) and spiked with ^13^C, ^15^N–labeled amino acids (algal amino acids ^13^C, ^15^N; Isotec, Miamisburg, OH, USA) at a concentration of 9 μg of the mix per milliliter. Amino acids were directly analyzed by LC-MS/MS as recently described (*54*). Amino acid concentrations in wheat leaves were determined using an AccQ-Tag kit (Waters, Milford, USA). Five hundred microliters of extract buffer (20 mM norleucine in MeOH) was added to 10 mg of dry and ground leaf powder. After the solution was thoroughly mixed for 15 min, 250 μl of chloroform and 500 of μl Milli-Q water were added. The mixture was centrifuged at 11,000*g* for 5 min at room temperature (20° to 23°C). Fifty microliters of supernatant was freeze dried into dry residue and then suspended with 50 μl of Milli-Q water. Five microliters of sample was briefly mixed with 35 μl of borate buffer and 10 μl of derivatization buffer from an AccQ-Tag Ultra Derivatization kit (Waters, USA) and then placed in a 55°C water bath for 10 min. Amino acid content was analyzed with a UPLC coupled to a UV detector and a QDa mass spectrometer (Waters, Milford, USA). Mixtures of 20 amino acids at 100, 50, 10, and 1 μg ml^−1^ were used as external standards for quantification. Soluble sugars, starch, and total soluble proteins were extracted and quantified as described previously (*38*).

### Callose induction

To determine callose deposition induced by chitosan, DIMBOA, DIMBOA-Glc, HDMBOA-Glc, and 4MO-I3M, callose abundance after chemical infiltration was analyzed from 15 randomly collected leaf segments from five different 10-day-old wheat seedlings and six 5-week-old *Arabidopsis* accession Columbia-0 (Col-0) plants per treatment. Each leaf was infiltrated with 500 μl of 0.2% (w/v) low viscous chitosan (Sigma, USA), which has a molecular weight of approximately 190 kDa. Chitosan was dissolved to 1% in 1% acetic acid and diluted to 0.2% by distilled water. DIMBOA, DIMBOA-Glc, and HDMBOA-Glc were dissolved to 80 μg/ml by 1.96% methanol and 0.04% acetic acid. 4MO-I3M (Phytoplan, Germany) was dissolved to 80 μg/ml by distilled water. Twenty-four hours after infiltration, leaf segments around the infiltration spots (approximately 4 cm^2^) were collected and destained by 96% ethanol to remove chlorophyll. Leaf segments with cell death symptoms were excluded. Destained leaf segments were stained with 70 mM phosphate buffer (pH 9.0) containing 0.01% aniline blue (Sigma, USA). Callose deposition was observed by epifluorescence microscopy (Zeiss, Germany), and callose spots were counted by eye. To determine callose deposition induced by aphid feeding, five aphid adults were placed in cages (2-cm diameter) on the leaves of 14-day-old wheat plants. After 7 days, all aphids were removed. Aphid-infested leaf segments were collected from 10 different plants per line. Leaf segment destaining and callose staining were performed as described above. As callose deposition following aphid infestation showed variable shapes and intensity, we first determined the total area of callose per plant (μm^2^) using Digimizer (MedCalc Software, Belgium). We then assessed the intensity of the staining on a relative scale from 1 (weak intensity) to 5 (strong intensity). Then, to obtain an integrated measure of callose induction, we multiplied intensity with callose area for each plant individually.

### Transcriptome sequencing and analysis

To identify herbivore-induced *OMT* genes in wheat, we sequenced the transcriptomes of three *S. littoralis*–undamaged and three *S. littoralis*–damaged wheat seedlings using Illumina HiSeq 2500. Total RNA was extracted from leaf material as described above, TruSeq RNA–compatible libraries were prepared, and PolyA enrichment was performed before sequencing the six transcriptomes on an IlluminaHiSeq 2500 with 21 Mio reads per library, 100 base pairs, paired end. Trimming of the obtained Illumina reads and mapping to the wheat gene model version 2.2 (*29*) were performed with the program CLC Genomics Workbench (Qiagen Bioinformatics) (mapping parameters: length fraction, 0.7; similarity fraction, 0.9; max number of hits, 25). Empirical analysis of digital gene expression (EDGE) implemented in the program CLC Genomics Workbench was used for gene expression analysis. A de novo transcriptome based on the Illumina reads from *S. littoralis*–damaged wheat leaves (~63 Mio reads in total) was constructed using the CLC Genomics Workbench (word size, auto; bubble size, 200) and was used for remapping with the same parameters as described above. Raw reads were deposited in the National Center for Biotechnology Information Sequence Read Archive under the accession no. SRP148745.

### Sequence analysis and tree reconstruction

OMTs were identified using a BLASTP analysis with maize BX7 as query and all available Poaceae protein datasets in Phytozome 12.1 (https://phytozome.jgi.doe.gov) as template. Genes with open reading frames >1000 nucleotides were considered as “full length” and used for phylogenetic analysis. Multiple sequence alignments were computed using the MUSCLE codon algorithm implemented in MEGA6 (*55*). On the basis of these alignments, trees were reconstructed with MEGA6 using a maximum likelihood algorithm. Codon positions included were 1st + 2nd + 3rd + noncoding. All positions with <90% site coverage were eliminated. Ambiguous bases were allowed at any position. A bootstrap resampling analysis with 1000 replicates was performed to evaluate the topology of the generated trees. A substitution model test was performed with MEGA6 to identify the best-fit substitution model for each dataset. The substitution models used for tree reconstructions are indicated in the respective figure legends.

### Cloning and expression of *TaBx10*

The complete open reading frame of *TaBx10* was amplified from cDNA with the primers ATGGTACGTCTCAGCGCATGCCGGCCGCGCAGCACAT (forward) and ATGGTACGTCTCATATCAAGGGTATACTTCGATAATTGATCGA (reverse) and inserted as Bsm BI fragment into the vector pASK-IBA37plus (IBA-GmbH, Göttingen), which allows the expression of *TaBx10* in *E. coli* NEB 10-beta cells (New England Biolabs) as N-terminal fusion protein. For expression, liquid cultures of bacteria harboring the expression construct were grown at 37°C and 220 rpm in lysogeny broth medium to an OD_600_ (optical density at 600 nm) of 0.6 to 0.8. Anhydrotetracycline was added to a final concentration of 200 μg liter^−1^, and the cultures were incubated for 20 hours at 18°C and 220 rpm. The cells were sedimented for 10 min at 5000*g* and 4°C. For breaking up the cells, the pellet was resuspended in ice-cold 4 ml of 50 mM tris-HCl (pH 8.0) containing 0.5 M NaCl, 20 mM imidazole, 20 mM 2-mercaptoethanol, and 10% glycerol and subsequently subjected to ultrasonication (4 × 20 s; Bandelin UW2070). The debris was separated by centrifugation for 20 min at 16,100*g* and 4°C. The N-terminal His-tagged TaBX10 was purified using Ni-NTA spin columns (Qiagen) according to the manufacturer’s instructions. The purified proteins were eluted with 50 mM tris-HCl (pH 8.0) containing 0.5 M NaCl, 250 mM imidazole, and 10% glycerol. The salt was removed by gel filtration using Illustra NAP-5 columns (GE Healthcare), and the protein was redissolved in 50 mM tris-HCl (pH 7.0) containing 10% glycerol.

### OMT enzyme assays

OMT activity of TaBX10 was tested using enzyme assays containing 0.5 mM dithiothreitol, 0.5 mM *S*-adenosyl-l-methionine, substrate (DIMBOA-Glc + DIM_2_BOA-Glc; 25 μg ml^−1^), and 35 μl of desalted enzyme in a volume of 100 μl. The assays were incubated overnight in glass vials at 25°C at 300 rpm using a ThermoMixer comfort 5355 (Eppendorf). The reaction was stopped with 1 volume 100% methanol and centrifuged for 5 min at 5000*g*. Product formation was monitored by the analytical methods described above.

### Insect bioassays

For caterpillar performance assays, 14-day-old wheat seedlings were infested with five second-instar *S. littoralis* larvae. The whole plants were covered by breathable cellophane bags (Celloclair AG, Switzerland) to prevent larvae from escaping. After 11 days, caterpillar weight gain (mg) was measured by microbalance (Mettler Toledo, USA). The leaf consumption (cm^2^) was calculated by Digimizer software (MedCalc Software, Belgium).

For caterpillar preference assays, one *ZmBx12*-overexpressing wheat plant and one wild-type plant were sown together in the same pot. Five second-instar *S. littoralis* larvae were put in the middle of the pot. Each pot was covered with transparent plastic film (Kodak, USA) to prevent larvae from escaping. The number of larvae feeding on each plant was recorded every 2 days for 8 days.

For aphid performance assays, 14-day-old wheat seedlings were infested with five adult *S. avenae* aphids. The whole seedling was covered with a breathable cellophane bag. Aphid progenies on the whole plants were counted during 11 days at regular time intervals.

To measure the effect of caterpillar feeding on aphid progeny production, five second-instar *S. littoralis* larvae were introduced onto the second leaf of 14-day-old wheat seedlings in clip cages. *S. littoralis* larvae were removed from plants after 24 hours. Five adult aphids were confined on the same leaves using clip cages for 7 days, and total aphid progenies were counted at the end.

### Pathogen bioassays

For the wheat leaf rust infection assay, 12-day-old wheat seedlings were infected with the virulent isolate 90035. After incubation for 24 hours in the dark, the whole seedlings were covered with permeable plastic hoods until the end of the experiment. Disease symptoms were assessed 11 days after inoculation.

For the wheat powdery mildew infection assay, infection tests were performed on detached segments of the first leaves of 9-day-old wheat seedlings, inoculated with the virulent isolates 96224 and JIM2. Disease levels were evaluated 6 days later by measuring the percentage of infected leaf area.

### Temporal effect of local induction by *S. littoralis* larvae and *S. avenae* on benzoxazinoids

Three second-instar *S. littoralis* larvae were attached in the second leaf of 14-day-old wheat seedlings by the clip cage and fed for 24 hours. After 24 hours, caterpillars were removed from the plants. Small leaf segments adjacent to the feeding sites were collected at several time points over a period of 7 days. After weighing, leaf segments were stored in the lysis tube containing ceramic beads and flash frozen with liquid nitrogen. The benzoxazinoid extraction buffer was added into the lysis tube (1 mg, 10 μl). Plant tissue was ground thoroughly by SpeedMill Plus (Analytik Jena, Germany) and then centrifuged at 13,200*g* for 20 min at 4°C twice to remove the pellet. Benzoxazinoids were analyzed as described above. To determine benzoxazinoid induction by aphids, five *S. avenae* adults were placed in cages (2-cm diameter) on the leaves of 14-day-old wheat plants. After 7 days, all aphids were removed. Aphid-infested leaf segments were collected from four to six different plants per line and analyzed as described above.

### Statistical analyses

Data were analyzed by ANOVA followed by Holm-Sidak post hoc tests implemented in Sigma Plot 13.0. Normality and error variance were determined, and datasets were log_10_ transformed to meet assumptions of ANOVAs where necessary.

## Supplementary Material

http://advances.sciencemag.org/cgi/content/full/4/12/eaat6797/DC1

## SUPPLEMENTARY MATERIALS

Supplementary material for this article is available at http://advances.sciencemag.org/cgi/content/full/4/12/eaat6797/DC1 Fig. S1. Phenotyping of *ZmBx12*-overexpressing plants I. Fig. S2. Phenotyping of *ZmBx12*-overexpressing plants II. Fig. S3. HDMBOA-Glc and MBOA levels upon DIMBOA and DIMBOA-Glc infiltration. Fig. S4. Specificity of benzoxazinoid- and glucosinolate-induced callose deposition. Fig. S5. Impact of DIMBOA-Glc *O*-methylation on wheat pathogen resistance. Fig. S6. Aphids do not induce benzoxazinoids in wheat leaves. Fig. S7. Identification of DIMBOA-Glc OMT candidate genes. Fig. S8. Phylogenetic tree of maize *OMT* genes similar to *Bx7* and wheat *OMT* genes that were found to be up-regulated after herbivory in wheat seedlings (RNA sequencing). Fig. S9. Sequence comparison of maize BX7 and BX10 with herbivore-induced OMT proteins from wheat. Fig. S10. Phylogenetic tree of maize and wheat *OMT* genes similar to *Bx7*. Fig. S11. Identification of *TaBx10* as a functional DIMBOA-Glc OMT. Fig. S12. No influence of *ZmBx12* overexpression on *TaBx10* expression. Fig. S13. Phylogenetic tree of Poaceae *OMT* genes similar to *Bx7*. Fig. S14. Phylogenetic tree of maize, wheat, and *Arabidopsis OMT* genes similar to *Bx7*. Table S1. Wheat *OMT* genes up-regulated after herbivory (RNA sequencing).
