# Supplementary material for: Convergent evolution of a metabolic switch between aphid and caterpillar resistance in cereals
Source: Sci Adv. 2018 Dec 5;4(12):eaat6797. doi: 10.1126/sciadv.aat6797 (PMC6281429; doi:10.1126/sciadv.aat6797)
Supplement: http://advances.sciencemag.org/cgi/content/full/4/12/eaat6797/DC1 [file supp_4_12_eaat6797__index.html]

Science Advances | Science Advances

## Supplementary Materials

**This PDF file includes:**

- Fig. S1. Phenotyping of *ZmBx12*-overexpressing plants I.
- Fig. S2. Phenotyping of *ZmBx12*-overexpressing plants II.
- Fig. S3. HDMBOA-Glc and MBOA levels upon DIMBOA and DIMBOA-Glc infiltration.
- Fig. S4. Specificity of benzoxazinoid- and glucosinolate-induced callose deposition.
- Fig. S5. Impact of DIMBOA-Glc *O*-methylation on wheat pathogen resistance.
- Fig. S6. Aphids do not induce benzoxazinoids in wheat leaves.
- Fig. S7. Identification of DIMBOA-Glc OMT candidate genes.
- Fig. S8. Phylogenetic tree of maize *OMT* genes similar to *Bx7* and wheat *OMT* genes that were found to be up-regulated after herbivory in wheat seedlings (RNA sequencing).
- Fig. S9. Sequence comparison of maize BX7 and BX10 with herbivore-induced OMT proteins from wheat.
- Fig. S10. Phylogenetic tree of maize and wheat *OMT* genes similar to *Bx7*.
- Fig. S11. Identification of *TaBx10* as a functional DIMBOA-Glc OMT.
- Fig. S12. No influence of *ZmBx12* overexpression on *TaBx10* expression.
- Fig. S13. Phylogenetic tree of Poaceae *OMT* genes similar to *Bx7*.
- Fig. S14. Phylogenetic tree of maize, wheat, and *Arabidopsis OMT* genes similar to *Bx7*.
- Table S1. Wheat *OMT* genes up-regulated after herbivory (RNA sequencing).

Download PDF

**Files in this Data Supplement:**

- Adobe PDF - aat6797\_SM.pdf
