# Supplementary material for: Convergent evolution of a metabolic switch between aphid and caterpillar resistance in cereals
Source: Sci Adv. 2018 Dec 5;4(12):eaat6797. doi: 10.1126/sciadv.aat6797 (PMC6281429; doi:10.1126/sciadv.aat6797)
Supplement: http://advances.sciencemag.org/cgi/content/full/4/12/eaat6797/DC1 [file aat6797_SM.pdf]

Science Advances
AAAS

advances.sciencemag.org/cgi/content/full/4/12/eaat6797/DC1

Supplementary Materials for

Convergent evolution of a metabolic switch between aphid and caterpillar resistance in cereals

B. Li, C. Förster, C. A. M. Robert, T. Züst, L. Hu, R. A. R. Machado, J.-D. Berset, V. Handrick, T. Knauer, G. Hensel, W. Chen, J. Kumlehn, P. Yang, B. Keller, J. Gershenzon, G. Jander, T. G. Köllner*, M. Erb*

*Corresponding author. Email: matthias.erb@ips.unibe.ch (M.E.); koellner@ice.mpg.de (T.G.K.)

Published 5 December 2018, *Sci. Adv.* **4**, eaat6797 (2018)
DOI: 10.1126/sciadv.aat6797

This PDF file includes:

Fig. S1. Phenotyping of *ZmBx12*-overexpressing plants I.
Fig. S2. Phenotyping of *ZmBx12*-overexpressing plants II.
Fig. S3. HDMBOA-Glc and MBOA levels upon DIMBOA and DIMBOA-Glc infiltration.
Fig. S4. Specificity of benzoxazinoid- and glucosinolate-induced callose deposition.
Fig. S5. Impact of DIMBOA-Glc *O*-methylation on wheat pathogen resistance.
Fig. S6. Aphids do not induce benzoxazinoids in wheat leaves.
Fig. S7. Identification of DIMBOA-Glc OMT candidate genes.
Fig. S8. Phylogenetic tree of maize *OMT* genes similar to *Bx7* and wheat *OMT* genes that were found to be up-regulated after herbivory in wheat seedlings (RNA sequencing).
Fig. S9. Sequence comparison of maize BX7 and BX10 with herbivore-induced OMT proteins from wheat.
Fig. S10. Phylogenetic tree of maize and wheat *OMT* genes similar to *Bx7*.
Fig. S11. Identification of *TaBx10* as a functional DIMBOA-Glc OMT.
Fig. S12. No influence of *ZmBx12* overexpression on *TaBx10* expression.
Fig. S13. Phylogenetic tree of Poaceae *OMT* genes similar to *Bx7*.
Fig. S14. Phylogenetic tree of maize, wheat, and *Arabidopsis* *OMT* genes similar to *Bx7*.
Table S1. Wheat *OMT* genes up-regulated after herbivory (RNA sequencing).

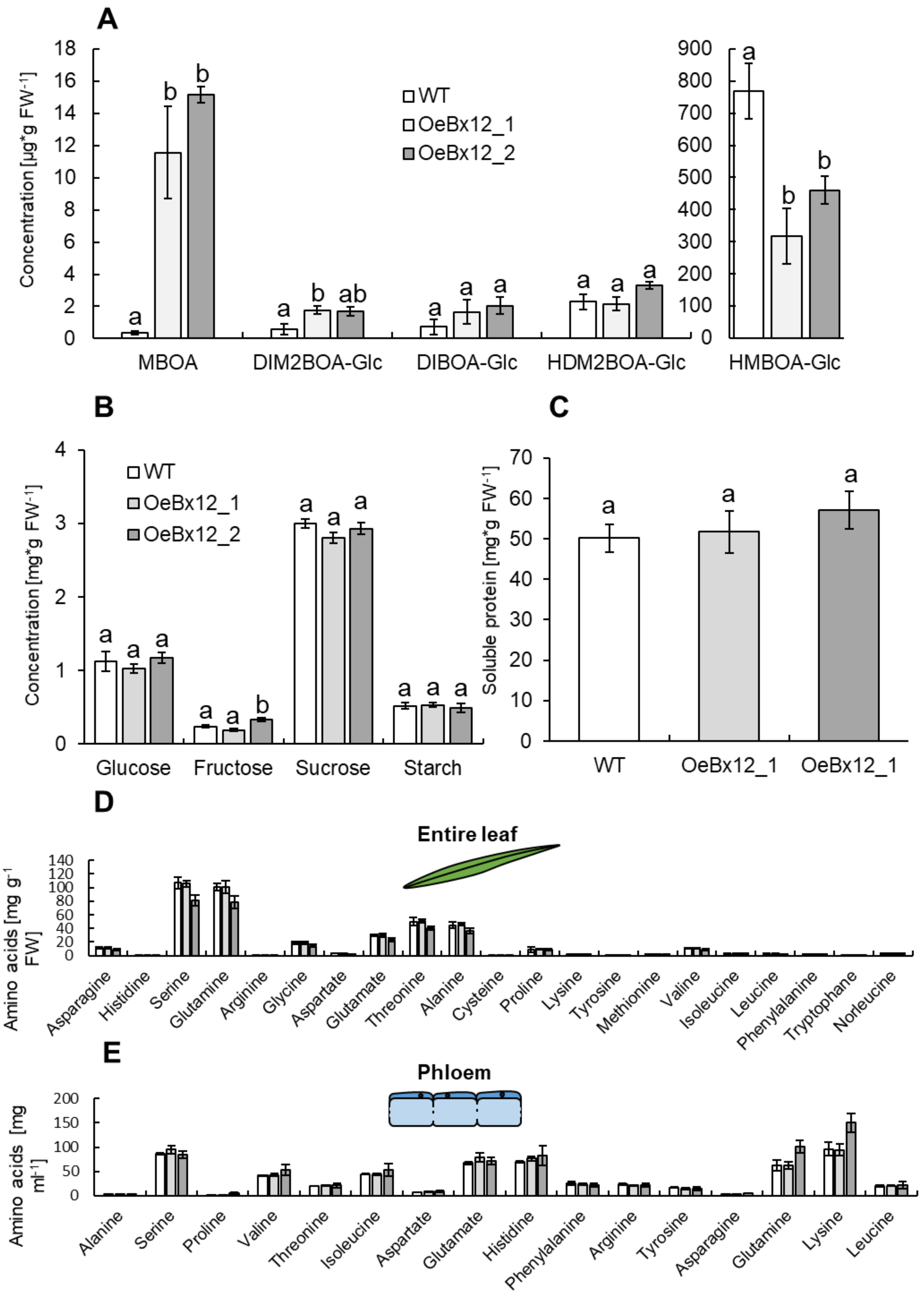

Fig. S1. Phenotyping of *ZmBx12*-overexpressing plants I. Concentrations of benzoxazinoids (**A**), sugars and starch (**B**), soluble protein (**C**), and free amino acids (**D**,**E**) in wild type (WT) and transgenic lines are shown. Different letters indicate significant differences between wheat lines (ANOVA followed by Holm-Sidak Post-Hoc tests, p<0.05).

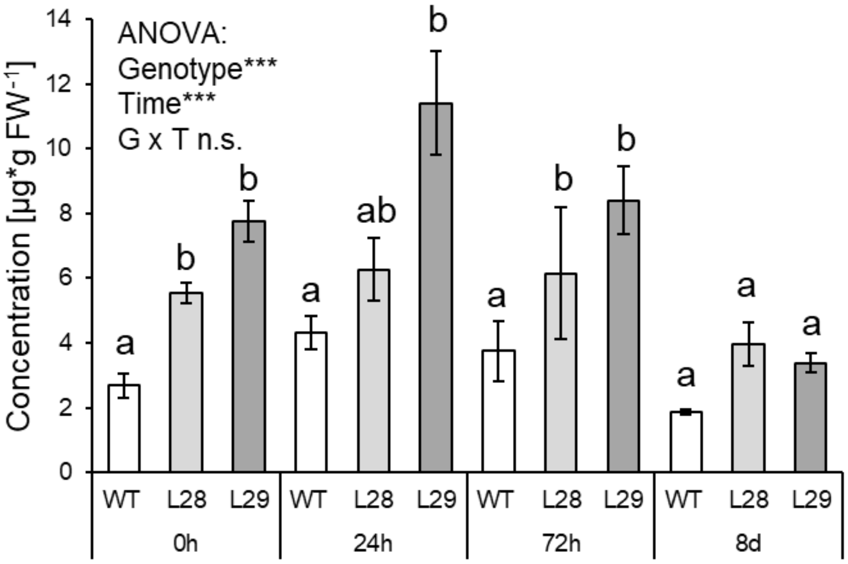

Fig. S2. Phenotyping of *ZmBx12*-overexpressing plants II. Concentrations of ferulic acid in wild type (WT) and transgenic lines in non-induced plants (0h) and *S. littoralis*-induced plants at different time points are shown. Chlorogenic acid, caffeic acid, coumaric acid and sinapic acid were below the limit of detection. Significance levels for Two-Way ANOVA factors are shown (*p<0.05; **p<0.01; ***p<0.001). Letters indicate significant differences between lines within time points (ANOVA followed by Holm-Sidak Post-Hoc tests, p<0.05). N.s. not significant.

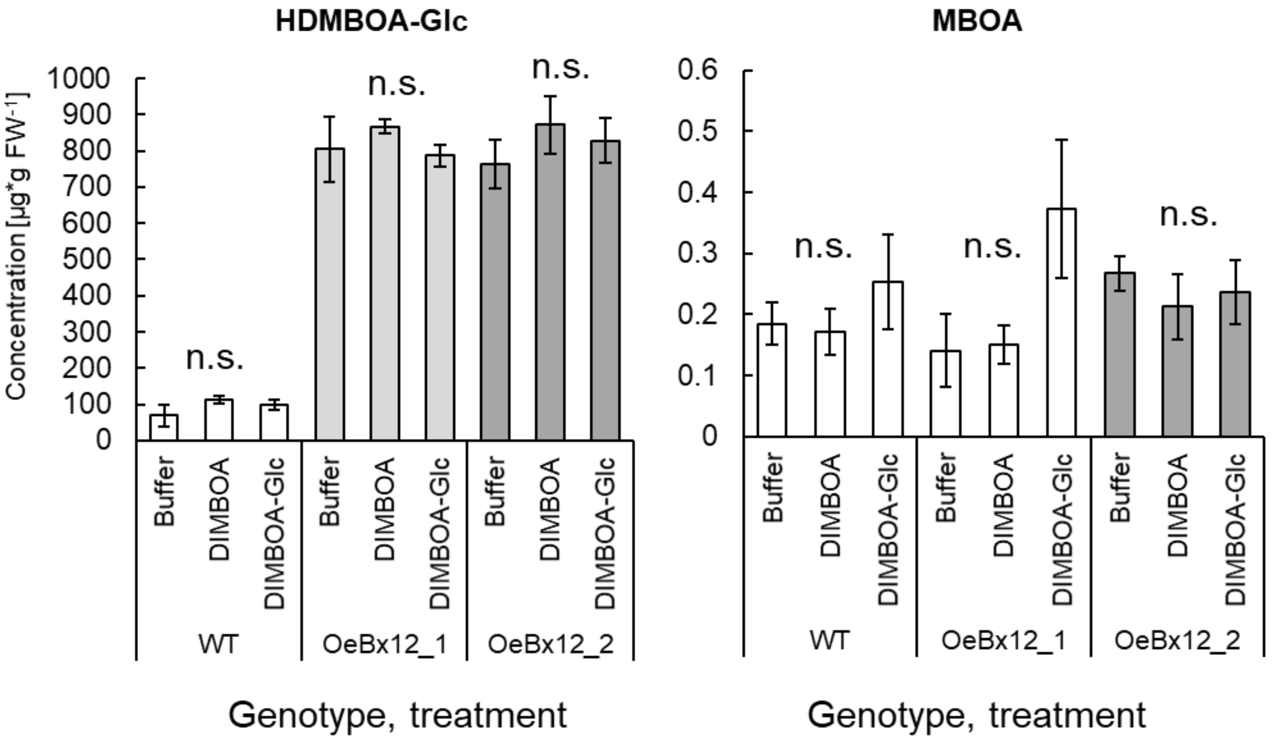

Fig. S3. HDMBOA-Glc and MBOA levels upon DIMBOA and DIMBOA-Glc infiltration. No significant differences between treatments within genotypes were detected.

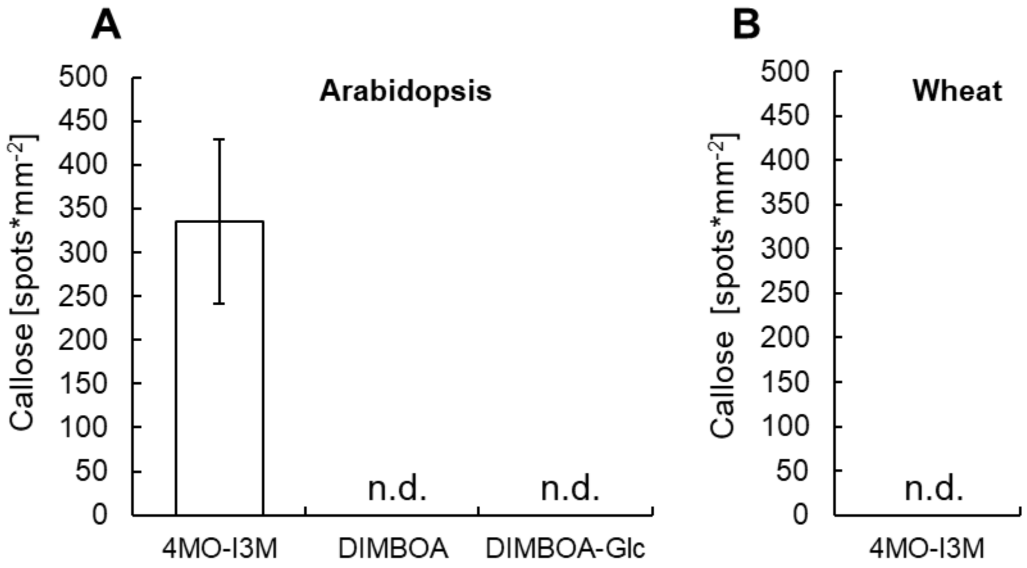

Fig. S4. Specificity of benzoxazinoid- and glucosinolate-induced callose deposition. Callose deposition in wheat (**A**) and *Arabidopsis thaliana* leaves (**B**) infused with the glucosinolate 4MO-l3M or the benzoxazinoids DIMBOA and DIMBOA-Glc (n=6). N.d. not detected.

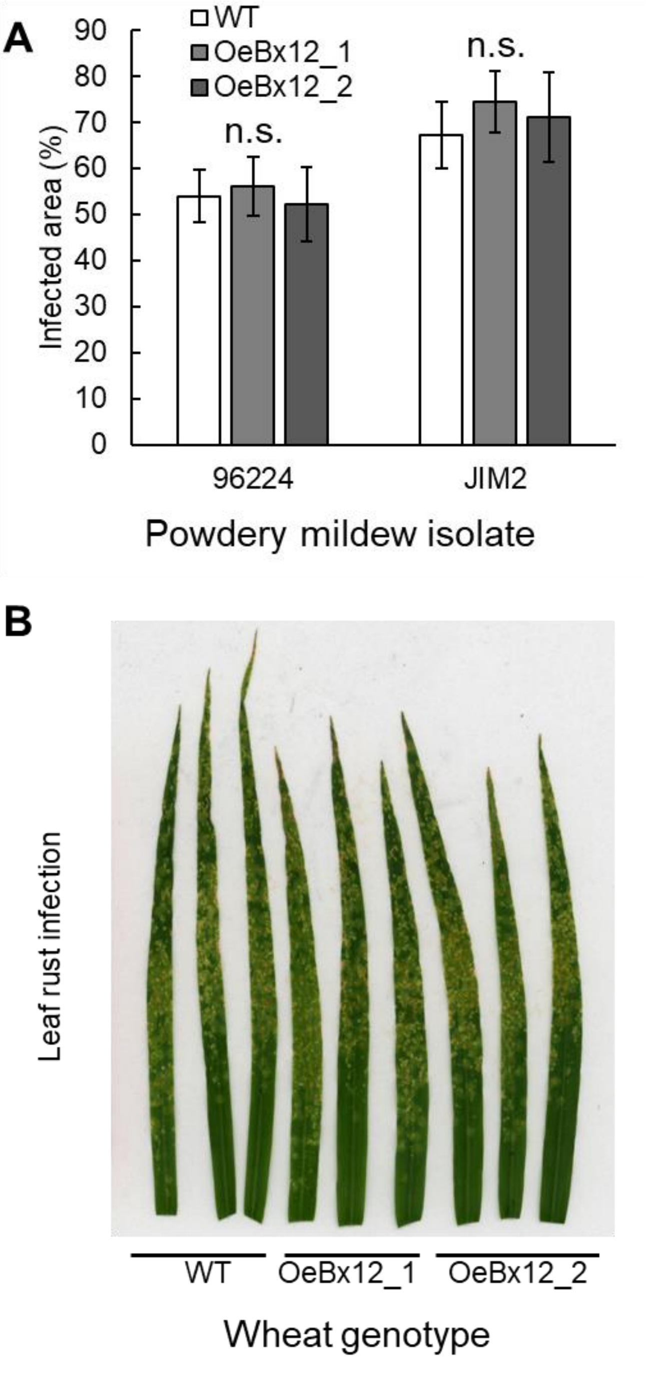

Fig. S5. Impact of DIMBOA-Glc *O*-methylation on wheat pathogen resistance. (**A**) infection severity caused by two powdery mildew (*Blumeria graminis* f. sp. tritici) isolates. No differences between wild type (WT) and transgenic lines were found (ANOVA followed by Holm-Sidak Post-Hoc tests, $p>0.05$). (**B**) representative photographs of leaf rust (*Puccinia recondita* f.sp.tritici) infested wheat leaves. Visual inspection revealed no differences in infection severity between the different lines. Photo credit for (B): Ping Yang, University of Zürich.

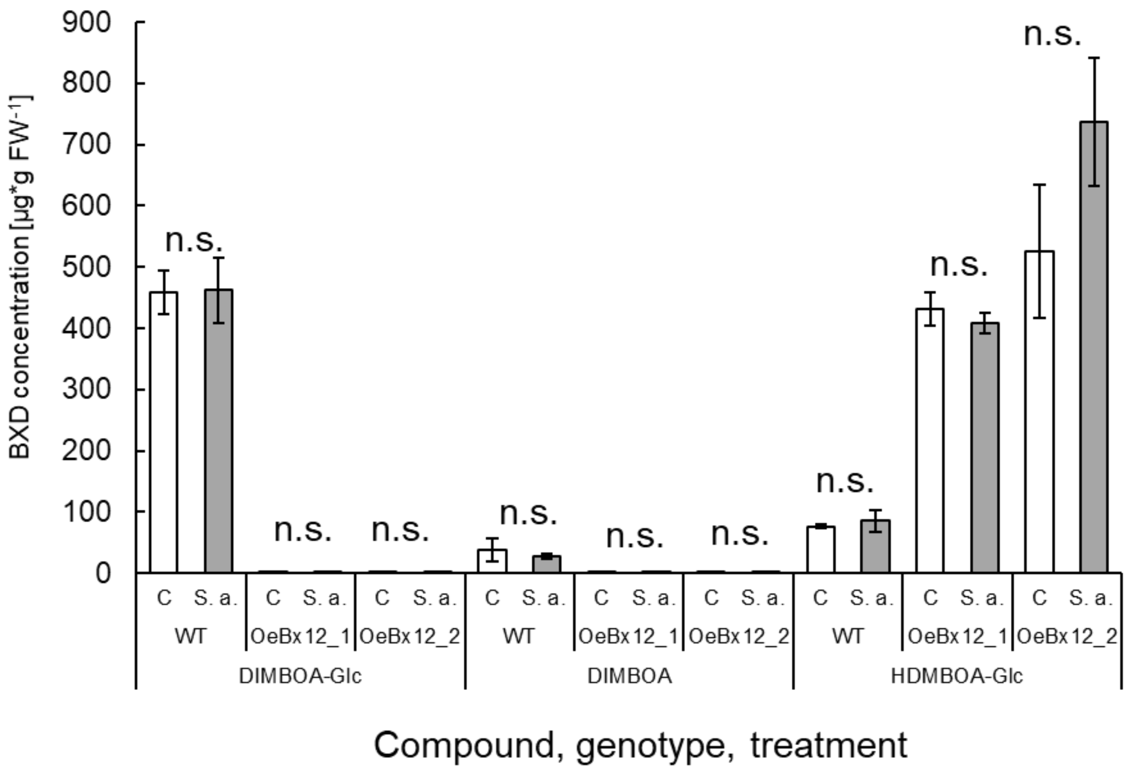

Fig. S6. Aphids do not induce benzoxazinoids in wheat leaves. Major benzoxazinoids in wild type (WT) and *ZmBx12* overexpressing leaves of control plants (C) and plants infested with *Sitobion avenae* aphids (S.a.). No significant differences between treatments within genotypes were detected.

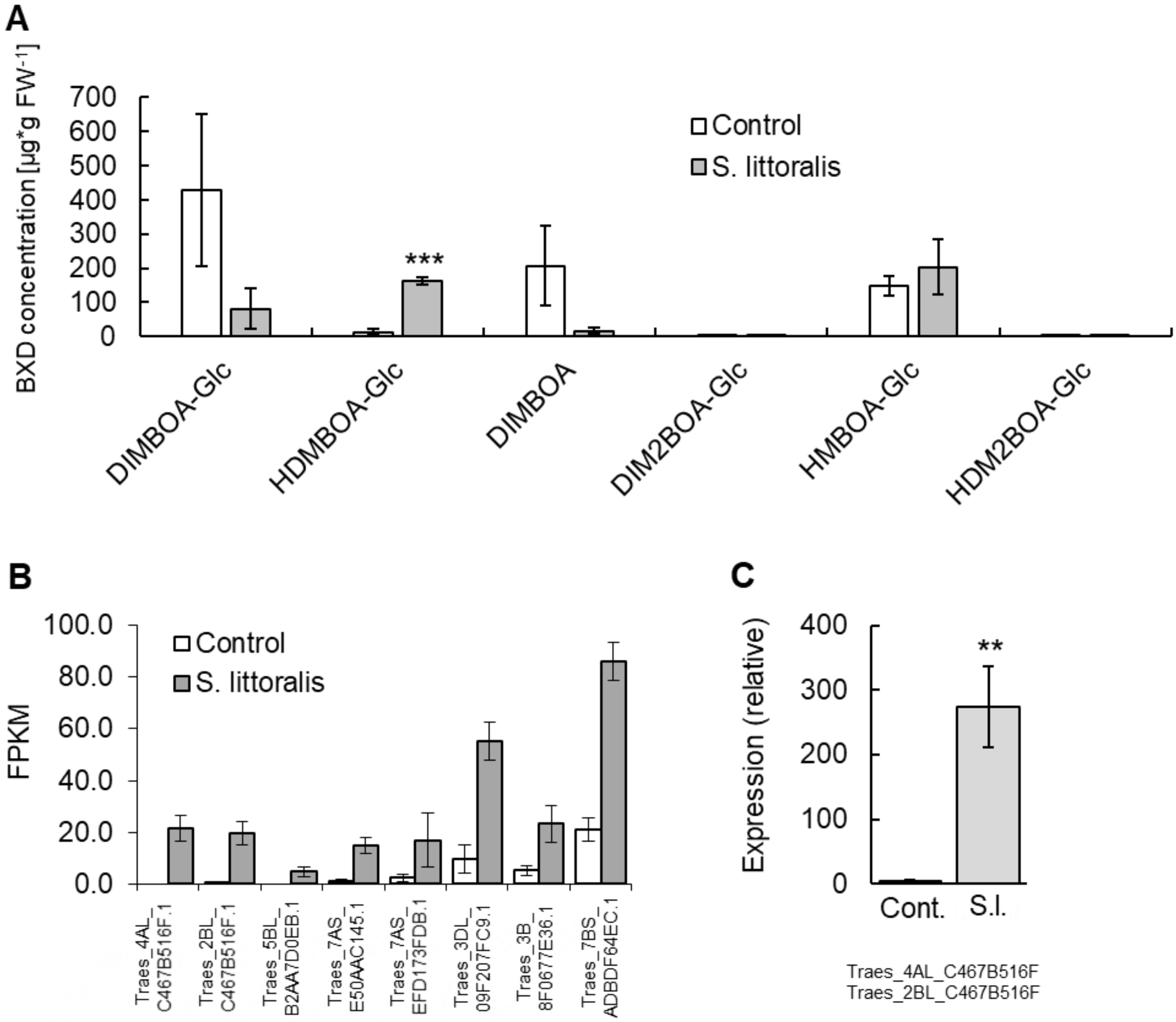

Fig. S7. Identification of DIMBOA-Glc OMT candidate genes. (A) Benzoxazinoid levels in control and *S. littoralis* induced wheat leaves (n=3). (B) The transcriptomes of the same samples were sequenced, and the obtained sequences were mapped to the primary gene models of the *T. aestivum* v2.2 genome. Differential gene expression was analyzed using the EDGE test implemented in the CLC genomics workbench package. Genes with a fold change > 3.5 and $P < 0.001$ were considered as upregulated. Means and SE are shown (n=3). (C) qRT-PCR analysis of Traes 4AL C467B516F/Traes 2BL C467B516F gene expression in herbivore-damaged wheat leaves. Means and SE are shown (n=6-7). Because 4AL C467B516F and Traes 2BL C467B516F share 100 % nucleotide identity, they could not be distinguished from each other in the qRT-PCR. Cont.: Control. S.l.: *Spodoptera littoralis.*

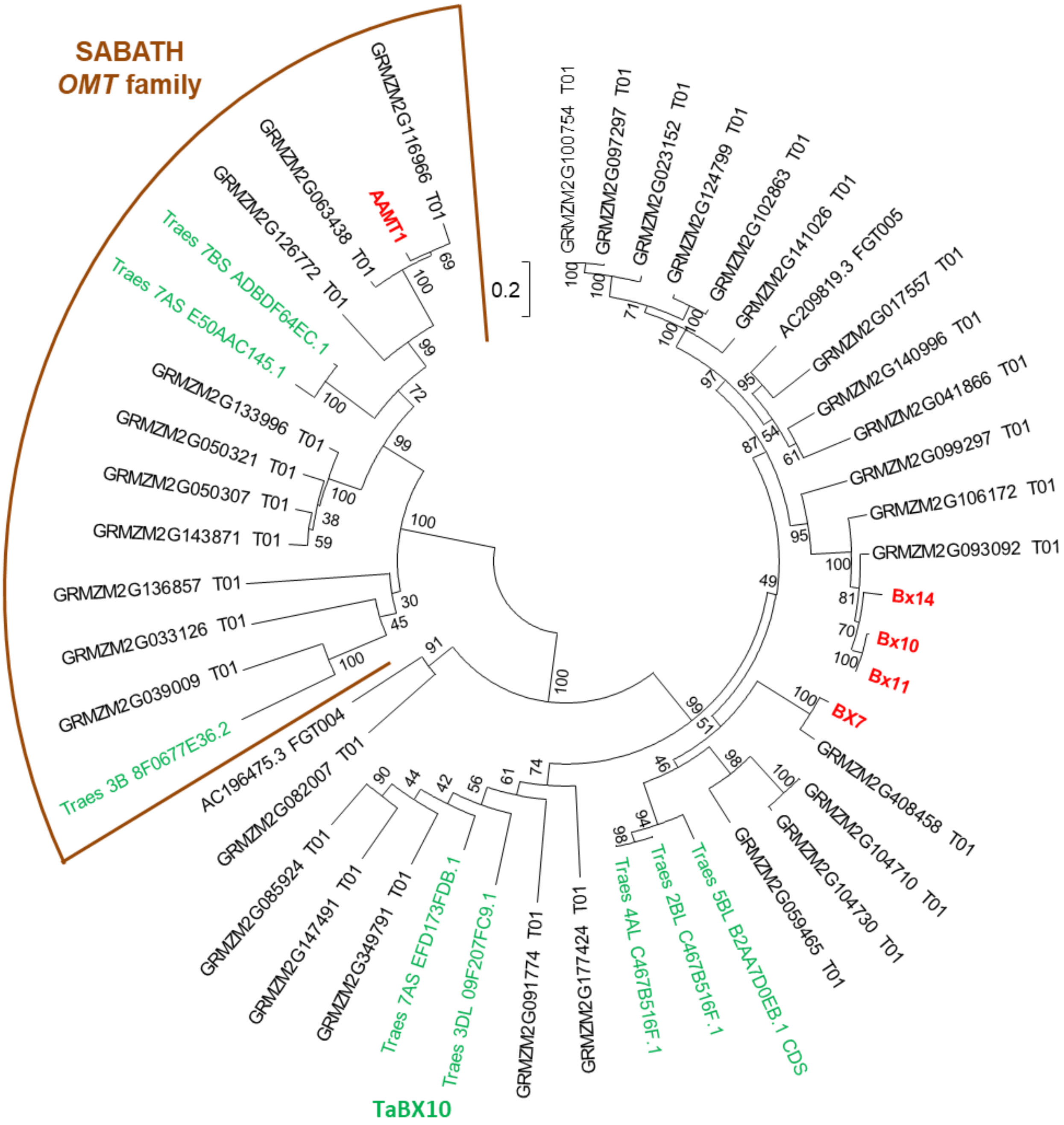

Fig. S8. Phylogenetic tree of maize *OMT* genes similar to *Bx7* and wheat *OMT* genes that were found to be up-regulated after herbivory in wheat seedlings (RNA sequencing). The tree was inferred by using the Maximum Likelihood method based on the General Time Reversible model. Bootstrap values (n = 1000 replicates) are shown next to each node. The tree is drawn to scale, with branch lengths measured in the number of substitutions per site. GRMZM, *Zea mays* genes (black); Traes, *Triticum aestivum* genes (green). Maize AAMT1 (anthranilic acid carboxyl methyltransferase 1) has been described as a member of the SABATH family (Köllner et al., 2010, Herbivore-Induced SABATH Methyltransferases of Maize That Methylate Anthranilic Acid Using *S*-Adenosyl-L-Methionine. *Plant Physiology* 153, 1795-1807).

```
BX7                    MGHQAQHGTD--------------------DTEELLAAHRQLWCHALGYVKSMALKCALDLRIPDTIDRCG-GSATLGELLAASEISASN
BX10                   MALMQES-----------------------SSQDLLQAHDELLHHSLCFAKSLALAVALDLRIPDAIHHHGAGGATLLQILAETALHPSK
Traes_2BL_C467B516F.1  MPAAQHIERDQDLAM---------------SSDELLQAQLELYHHGLAFVKSLALKAATDLRIPDAIHRRG-GAATLSELASDTGIHPTK
Traes_4AL_C467B516F.1  MPAAQHIERDQDLAM---------------SSDELLQAQLELYHHGLAFVKSLALKAATDLRIPDAIHRRG-GAATLSELASDTGIHPTK
Traes_5BL_B2AA7D0EB.1  GRSKKQVAASIPHSIDHTHTPCRPRSTSNETKTELLQAQLELYHHGLAFVKSLALKAATDLRIPDAIHRRG-GAATLSELASDTGIHPTK
Traes_7AS_E50AAC145.1  ------------------------------------------------------------------------------------------
Traes_7AS_EFD173FDB.1  ------------------------------------------------------------------------------------------
Traes_3DL_09F207FC9.1  ------------------------------------------------------------------------------------------
Traes_3B_8F0677E36.2   M-------------------------------------------------KPLIEEVITDLCSSTSTLLH--GKIVIADLGCSSG--PNA
Traes_7BS_ADBDF64EC.1  MKEASGVRMVTG------------------DGENSYAANSRLQEKAILETRPVLRKAIQEVCTSPSARR---STMVVADLGCSSG--PNT

BX7                    HDYLRRVMRTLTAMRIFAASH----------DPAKADDAAAISYQLTPASRLLVSSSSSVDDAAGASKENTTTPSILPNIAHLVRPNTIS
BX10                   LRALRRLMRVLTVTGIFSVVEQPPAGGGDDSTVHTSDDEAVVVYRLTAASRFLVSD-------------DVSTATLAPFVSLALQPIAAC
Traes_2BL_C467B516F.1  RSNLRRVMRVLTTTGVFSIVQGKGS------NDHAGDGAA--YYKLTRVSRLLV---------------ERSPHNLSPMVGTIVNTLCWT
Traes_4AL_C467B516F.1  RSNLRRVMRVLTTTGVFSIVQGKGS------NDHAGDGAA--YYKLTRVSRLLV---------------ERSPHNLSPMVGTIVNTLCWT
Traes_5BL_B2AA7D0EB.1  RSNLRRVMRVLTTTGVFSIVQGKGS------NDHAGD-----------------------------------------------------
Traes_7AS_E50AAC145.1  ------------------------------------------------------------------------------------------
Traes_7AS_EFD173FDB.1  ------------------------------------------------------------------------------------------
Traes_3DL_09F207FC9.1  ------------------------------------------------------------------------------------------
Traes_3B_8F0677E36.2   LALVSTAINAIHNHCL--QLQQPPPEICVLLNDLPDTDFNTVVKSLVTLRQS-----------------KNPVVVTGVAPGSFYERLFTS
Traes_7BS_ADBDF64EC.1  LSFISEVIGAVRSCTRKSEEERRAVEVQFFLNDLPGNDFNLVFRSLEQFENLSRK--------------DTPPYYVVGLPRSYYRKLFPS

BX7                    L-LFSMGEWMK----DESAASVSLYETVHRQGMWACVEDDAANRASFYESMDADTRLVMQAVVRRCPHVFDGIK-SLVDVGGGRGTAAAA
BX10                   PHALGISAWFRQEQHEPSPYGLAFRQTP---TIWEHADDVN---ALLNKGMAADSRFLMPIVLRECGETFRGID-SLVDVGGGHGGAAAT
Traes_2BL_C467B516F.1  S-LLKMPEWFTQGQEGESAQSHSLVQLANGCTFWDTTKVDG---GLFNDGMAADSRIAMKVLLKEHGGAFGEVKSSLVDIGGNHGATASA
Traes_4AL_C467B516F.1  S-LLKMPEWFTQGQEGESAQSHSLVQLANGCTFWDTTKVDG---GLFNDGMAADSRIAMKVLLKEHGGAFGEVKSSLVDIGGNHGATASA
Traes_5BL_B2AA7D0EB.1  ------------------------------------------------------------------------------------------
Traes_7AS_E50AAC145.1  ------------------------------------------------------------------------------------------
Traes_7AS_EFD173FDB.1  ------------------------------------------------------------------------------------------
Traes_3DL_09F207FC9.1  ------------------------------------------------------------------------------------------
Traes_3B_8F0677E36.2   NSLHVVCASNSLQWLSKAPEDLTRNRIP---AFNIDEHARREMLPMVREAYEQQFRKDFKLFLELRAKELVSGGRMVISLVGTRSDVIAS
Traes_7BS_ADBDF64EC.1  RSVHFFHSSYSLMWRSKVPEELSSCTHLNEGNIYIGKTTP----PMVIKLFQEQFQKDFELFLTLRFKELVSGGRMLLTFLGRKNEEMMT

BX7                    VVAAFPHIQRCTVMDLPHVVAEAPAGTAGLSFHGGDMFEHIPSADALMLKWILHDWDEDKCIKIMERCKEAIGGKEAGGKVIIIDTVLGS
BX10                   IAAAFPHL-KCSVLDLPHVVAGAPSDGNV-QFVAGNMFESIPPATAVFLKKTLHDWGDDECVKILKNCKQAISPRDAGGKVIILDVVVGY
Traes_2BL_C467B516F.1  VARAFPHL-KCSVLDLPHVVAAAPASDIL-TFVAGNMFEYVPPADAVLLKWILHDWKHEDCVKIMRRCKEAIPAKEAGGKVIIIDMVVGY
Traes_4AL_C467B516F.1  VARAFPHL-KCSVLDLPHVVAAAPASDIL-TFVAGNMFEYVPPADAVLLKWILHDWKHEDCVKIMRRCKEAIPAKEAGGKVIIIDMVVGY
Traes_5BL_B2AA7D0EB.1  ------------------------------------------------------------------------------------------
Traes_7AS_E50AAC145.1  ---------------------GRVEKEKLDSFNVPYYTPSVKEVRELINKSRLFD------IEHARLFESNWDPQDDSDGDVVLDYA-GS
Traes_7AS_EFD173FDB.1  --------------------------------------------------SVLHLWSDEDCIKILAQCKKAIPPRGAGGKVIVIDIVLSS
Traes_3DL_09F207FC9.1  -------------------------------------------------QLVLHWWCDEDCIKILAECKKSIPSREEGGKVIVIDIVVGH
Traes_3B_8F0677E36.2   EFSLFPGI--LAQILSVMVAEGVIDKAKFDSFYVPLHGPSIEEVREIIKEEGSFS------IKEMRVHDPTAE----------MNIALSS
Traes_7BS_ADBDF64EC.1  HGEVGTLYELVAESLLSLVLKGRVEKEKLDSFNVPYYTPSVKEVRELINKSRLFD------IEHARLFESNWDPQDDSDGDVVLDYA-GS

BX7                    RADDDDDDKTCRETYVLDLHILSFVNGAEREEHEWRRIFLAAGFRDYKITHTRG-IPSIIEVFP*
BX10                   KQ---SNIKHQETQVMFDLYMMA-VNGVERDEQEWKKIFTEAGFKDYKILPVIGDVSVIIEVYP*
Traes_2BL_C467B516F.1  PVTQPNHSK--EAQVLLDIYMMG-SDGMEREENEWSLIFSEAGFSDYKITPTNG-IRSIIEVYP*
Traes_4AL_C467B516F.1  PVTQPNHSK--EAQVLLDIYMMG-SDGMEREENEWSLIFSEAGFSDYKITPTNG-IRSIIEVYP*
Traes_5BL_B2AA7D0EB.1  -----------------------------------------------------------------
Traes_7AS_E50AAC145.1  GANVANCIRAVMEPLIVDHFGEDIID-------DLFVVFASIVAKHLEKAKAKYPIIVLSLKKAT
Traes_7AS_EFD173FDB.1  -----ASGLMLETHHLVDMLKLVMTRGRQRDEKDWSDIFMKAGFSEYKIFKKVG-ARAVIEVYP*
Traes_3DL_09F207FC9.1  -----SCEATYEPQVLADMLM--------------------------------------------
Traes_3B_8F0677E36.2   PSKFVNNLIALFEPIIVQHFGEVMDEFVRAAELHWSL--DVDGSLRQERARTSRAMLVVSLAKA*
Traes_7BS_ADBDF64EC.1  GANVANCIRAVLEPLIVDHFGEDIID-------DLFVVFASIVAKHLEKAKAKYPIIVLSLKKAM
```

Fig. S9. Sequence comparison of maize BX7 and BX10 with herbivore-induced OMT proteins from wheat. Amino acids identical in at least 5 out of 10 sequences are marked by black boxes and amino acids with similar side chains are marked by gray boxes. The genes encoding Traes_2BL_C467B516F.1 and Traes_4AL_C467B516F.1 have identical nucleotide sequences and were both designated as *TaBX10* (shown in red).

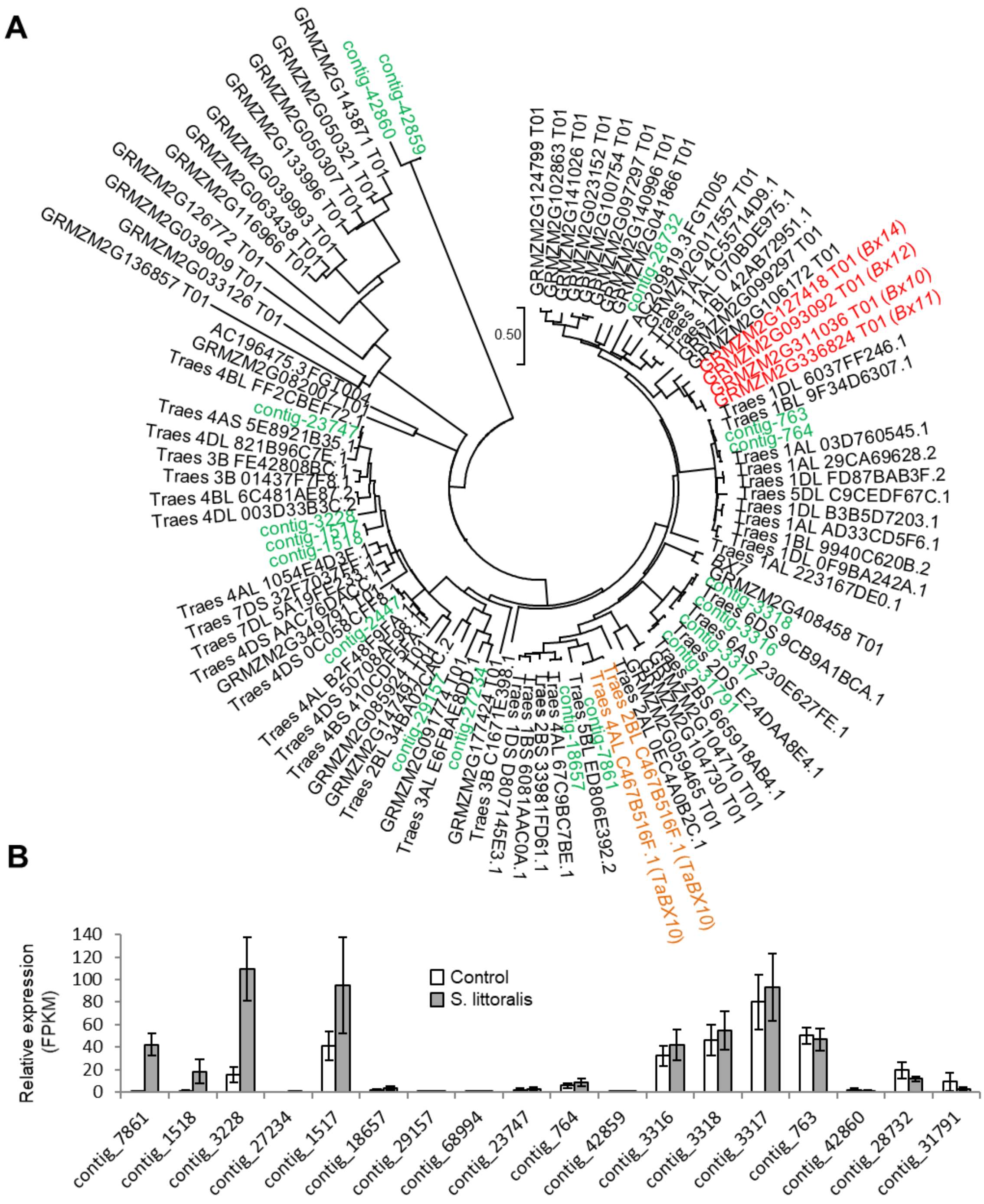

Fig. S10. Phylogenetic tree of maize and wheat *OMT* genes similar to *Bx7*. (**A**) and expression values of *OMT* contigs extracted from a *de novo* transcriptome of herbivore-damaged wheat leaves (**B**). OMTs were identified using a BLASTP analysis with maize BX7 as query and the maize and wheat protein datasets in Phytozome 12.1 (https://phytozome.jgi.doe.gov) as template. In addition, a *de novo* transcriptome of *Spodoptera littoralis* damaged wheat leaves was used as template for a TBLASTN analysis with *Bx10* as query. Genes with ORFs > 1000 nucleotides were considered as ‘full-length’ and used for phylogenetic analysis. Maize *Bx10,11,12,14*, wheat *TaBX10*, and wheat *OMT* contigs found in the *de novo* transcriptome are shown in red, brown, and green, respectively. The tree was inferred by using the Maximum Likelihood method based on the Tamura 3-parameter model. The tree is drawn to scale, with branch lengths measured in the number of substitutions per site. GRMZM, *Zea mays*; Traes, *Triticum aestivum*; contig, contig number in the *de novo* transcriptome.

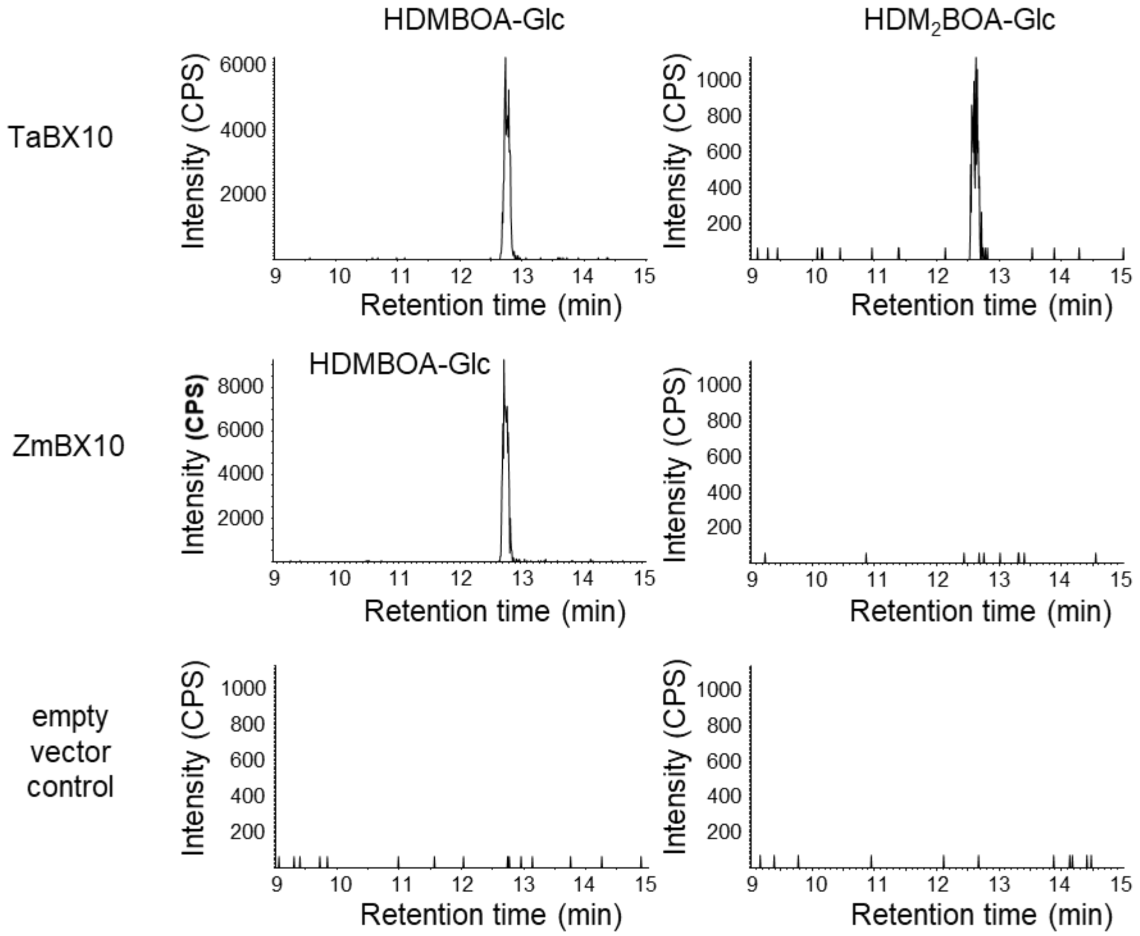

Fig. S11. Identification of *TaBx10* as a functional DIMBOA-Glc OMT. Recombinant Traes 4AL C467B516F (TaBX10) accepts DIMBOA-Glc and DIM$_2$BOA-Glc as substrate and produces HDMBOA-Glc and HDM$_2$BOA-Glc, respectively. The enzymes were heterologously expressed in *E. coli*. Purified recombinant proteins were incubated with a mixture of DIMBOA-Glc and DIM$_2$BOA-Glc as substrate and SAM as co-substrate. Enzyme products were analyzed using LC-MS/MS. Maize ZmBX10 was included as positive control and an *E. coli* strain expressing an empty vector was used as negative control.

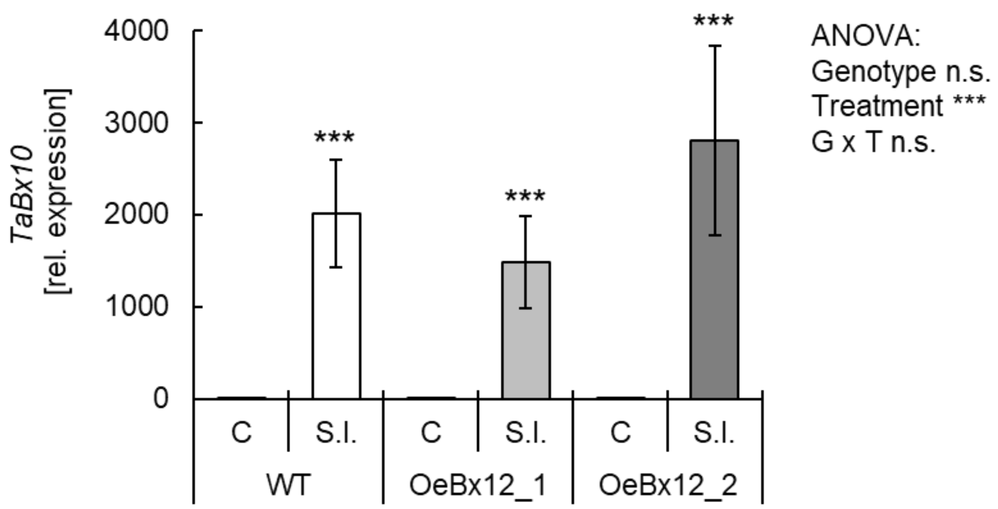

Fig. S12. No influence of *ZmBx12* overexpression on *TaBx10* expression. Relative expression of *TaBx10* in the leaves of WT and OeBx12 plants that were uninfested or infested with *S. littoralis* larvae for 24 h (*n*=5-6). Stars above bars indicate a significant difference between treatments within genotypes (Holm-Sidak Post-Hoc tests, *p<0.05; **p<0.01; ***p<0.001). Significance levels for ANOVA factors are shown on the right (*p<0.05; **p<0.01; ***p<0.001) C: Control. S.l.: *Spodoptera littoralis.*

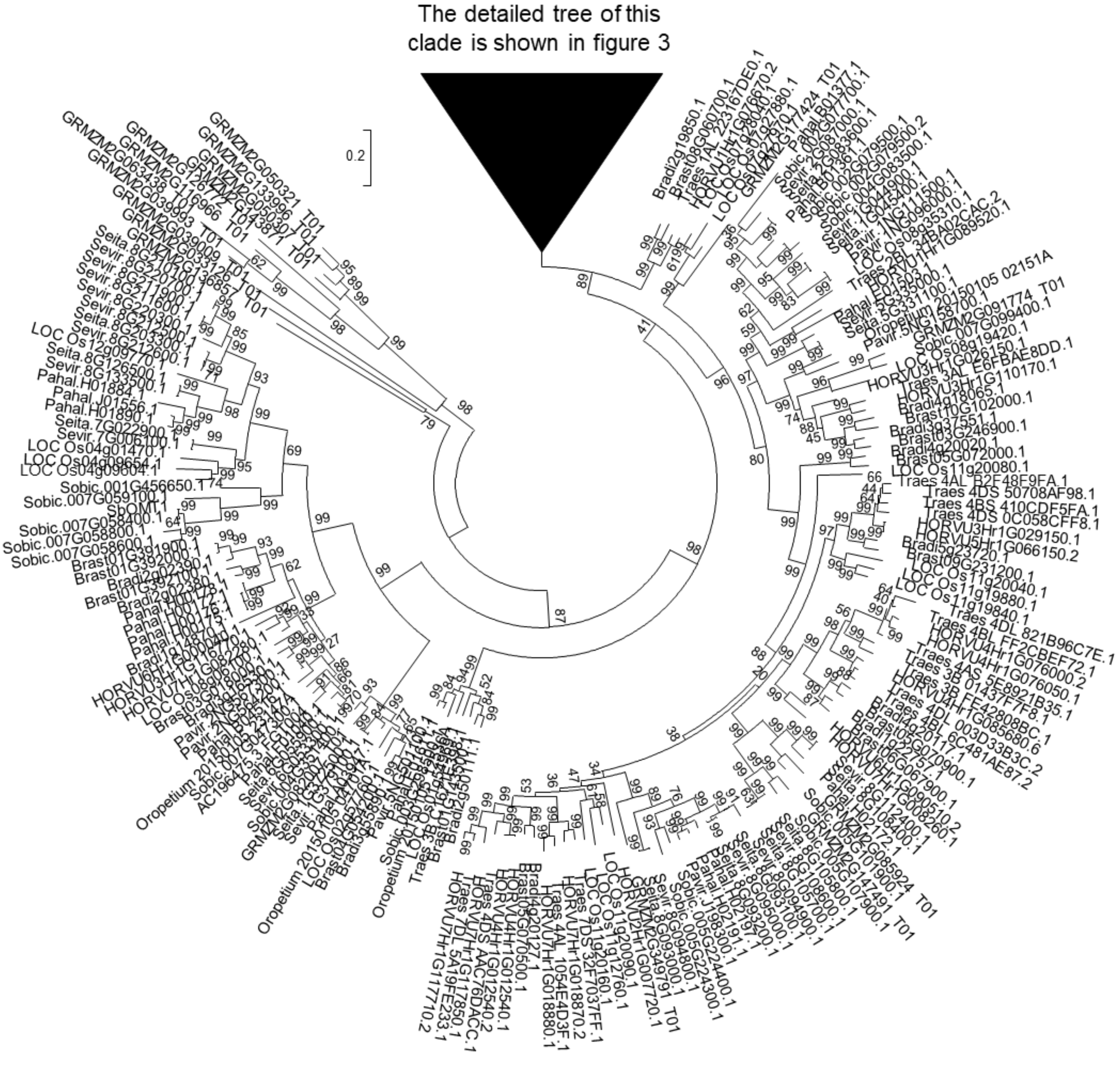

Fig. S13. Phylogenetic tree of Poaceae *OMT* genes similar to *Bx7*. OMTs were identified using a BLASTP analysis with maize BX7 as query and all available Poaceae protein datasets in Phytozome 12.1 (https://phytozome.jgi.doe.gov) as template. Genes with ORFs > 1000 nucleotides were considered as 'full-length' and used for dendrogram analysis. The tree was inferred by using the Maximum Likelihood method based on the Tamura 3-parameter model. Bootstrap values (n = 1000 replicates) are shown next to each node. The tree is drawn to scale, with branch lengths measured in the number of substitutions per site. GRMZM, *Zea mays*; Sobic, *Sorghum bicolor*; Sevir, *Setaria viridis*; Seita, *S. italica*; Pavir, *Panicum virgatum*; Pahal, *P. hallii*; Oropetium, *Oropetium thomaeum*; Traes, *Triticum aestivum*; HORVU, *Hordeum vulgare*; Bradi, *Brachypodium distachyon*; Brast, *B. stacei*; LOC Os, *Oryza sativa*.

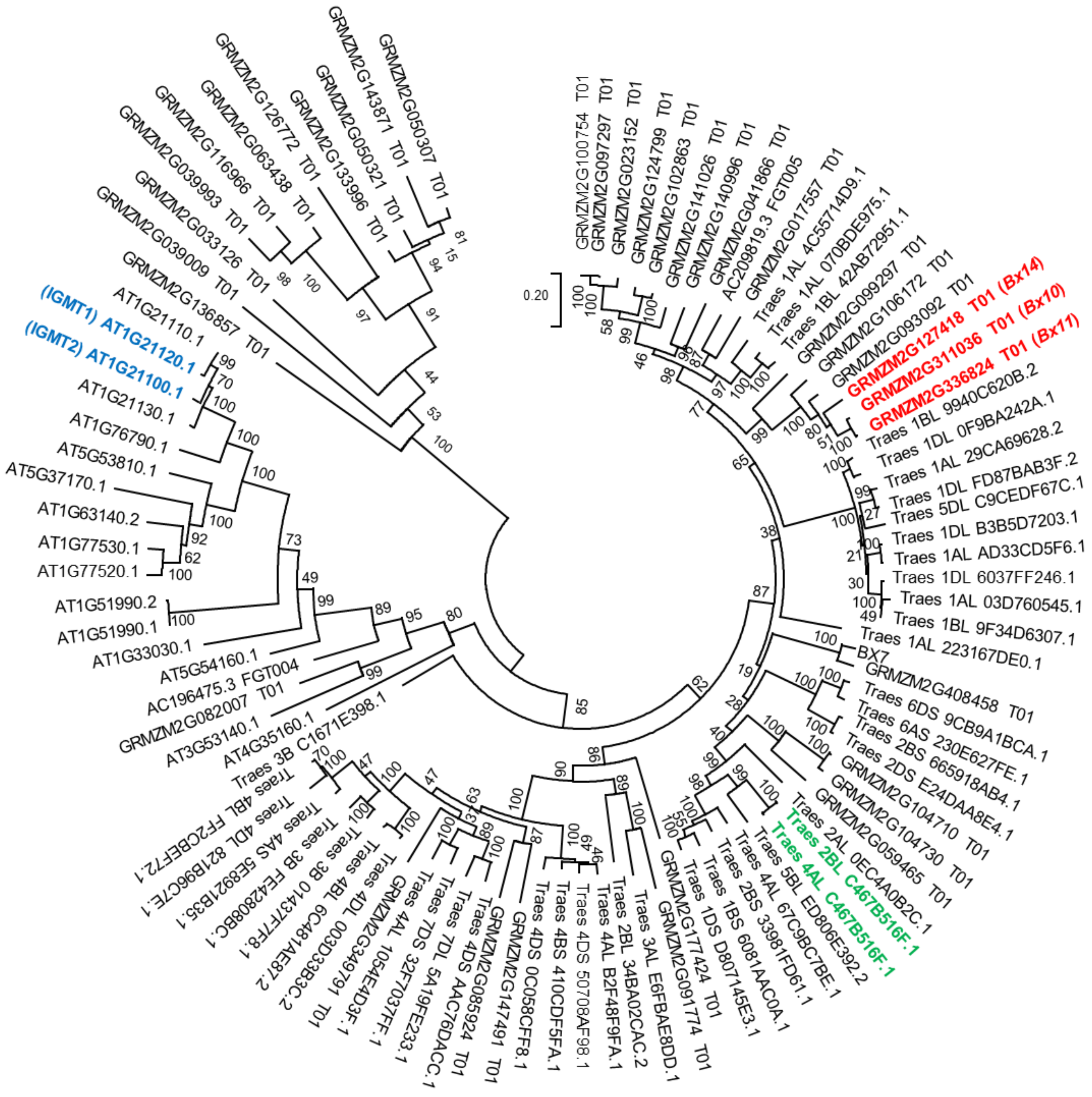

Fig. S14. Phylogenetic tree of maize, wheat, and *Arabidopsis OMT* genes similar to *Bx7*. OMTs were identified using a BLASTP analysis with maize BX7 as query and the maize, wheat, and *Arabidopsis thaliana* protein datasets in Phytozome 12.1 (https://phytozome.jgi.doe.gov) as template. Genes with ORFs > 1000 nucleotides were considered as 'full-length' and used for phylogenetic analysis. Maize *Bx10,11,14,* wheat *TaBX10*, and Arabidopsis IGMT1,2 are shown in red, green, and blue, respectively. The tree was inferred by using the Maximum Likelihood method based on the Tamura 3-parameter model. Bootstrap values (n = 1000 replicates) are shown next to each node. The tree is drawn to scale, with branch lengths measured in the number of substitutions per site. GRMZM, *Zea mays*; Traes, *Triticum aestivum*; AT, *Arabidopsis thaliana*.

Table S1. Wheat *OMT* genes up-regulated after herbivory (RNA sequencing). Wheat seedlings were subjected to herbivory by *Spodoptera littoralis*. The transcriptomes of three damaged and three undamaged plants were sequenced and compared to identify herbivory-induced *O*-methyltransferase genes. All identified *OMT* genes with a fold change > 3.0 and a *P*-value < 0.001 are shown.

Feature ID	EDGE test Fold change	EDGE test P-value	EDGE test Weighted difference	leaf ctr - 2644_A FPKM	leaf ctr - 2644_C FPKM	leaf ctr - 2644_B FPKM	leaf herb - 2644_E FPKM	leaf herb - 2644_D FPKM	leaf herb - 2644_F FPKM	leaf ctr - Means FPKM	leaf herb - Means FPKM	annotation
Traes_4AL_C467B516F.1	161.6	1E-13	1.58E-05	0.0	0.0	0.0	16.5	18.9	29.4	0.0	21.6	PTHR11746//PTHR11746:SF108 - O-METHYLTRANSFERASE
Traes_2BL_C467B516F.1	96.9	1E-11	1.44E-05	0.0	0.0	0.2	15.7	16.6	27.1	0.1	19.8	PTHR11746//PTHR11746:SF108 - O-METHYLTRANSFERASE
Traes_5BL_B2AA7D0EB.1	35.7	0.0001	3.42E-06	0.0	0.0	0.0	1.9	5.0	7.3	0.0	4.7	PTHR11746//PTHR11746:SF108 - O-METHYLTRANSFERASE
Traes_7AS_E50AAC145.1	11.5	5E-07	9.89E-06	2.1	0.6	0.3	14.1	10.9	19.4	1.0	14.8	PTHR31009//PTHR31009:SF16 - S-ADENOSYL-L-METHIONINE:CARBOXYL METHYLTRANSFERASE
Traes_7AS_EFD173FDB.1	6.3	0.0029	1.1E-05	2.5	0.0	4.4	0.8	19.8	30.4	2.3	17.0	2.1.1.240 - Trans-resveratrol di-O-methyltransferase / Resveratrol O-methyltransferase
Traes_3DL_09F207FC9.1	4.7	2E-07	3.19E-05	7.1	3.7	18.3	53.3	46.2	66.4	9.7	55.3	PTHR11746//PTHR11746:SF91 - O-METHYLTRANSFERASE
Traes_3B_8F0677E36.2	3.8	0.0002	1.28E-05	5.7	2.0	7.8	12.1	25.9	31.7	5.2	23.2	PTHR31009//PTHR31009:SF12 - S-ADENOSYL-L-METHIONINE:CARBOXYL METHYLTRANSFERASE
